# The α5 Nicotinic Acetylcholine Receptor Subunit Differentially Modulates α4β2^*^ and α3β4^*^ Receptors

**DOI:** 10.3389/fnsyn.2020.607959

**Published:** 2020-12-03

**Authors:** Petra Scholze, Sigismund Huck

**Affiliations:** Department of Pathobiology of the Nervous System, Center for Brain Research, Medical University of Vienna, Vienna, Austria

**Keywords:** nACh receptor, CHRNA5 polymorphism, subunit composition, heterologous expression, endogenous receptors, calcium

## Abstract

Nicotine, the principal reinforcing compound in tobacco, acts in the brain by activating neuronal nicotinic acetylcholine receptors (nAChRs). This review summarizes our current knowledge regarding how the α5 accessory nAChR subunit, encoded by the *CHRNA5* gene, differentially modulates α4β2^*^ and α3β4^*^ receptors at the cellular level. Genome-wide association studies have linked a gene cluster in chromosomal region 15q25 to increased susceptibility to nicotine addiction, lung cancer, chronic obstructive pulmonary disease, and peripheral arterial disease. Interestingly, this gene cluster contains a non-synonymous single-nucleotide polymorphism (SNP) in the human *CHRNA5* gene, causing an aspartic acid (D) to asparagine (N) substitution at amino acid position 398 in the α5 nAChR subunit. Although other SNPs have been associated with tobacco smoking behavior, efforts have focused predominantly on the D398 and N398 variants in the α5 subunit. In recent years, significant progress has been made toward understanding the role that the α5 nAChR subunit—and the role of the D398 and N398 variants—plays on nAChR function at the cellular level. These insights stem primarily from a wide range of experimental models, including receptors expressed heterologously in *Xenopus* oocytes, various cell lines, and neurons derived from human induced pluripotent stem cells (iPSCs), as well as endogenous receptors in genetically engineered mice and—more recently—rats. Despite providing a wealth of available data, however, these studies have yielded conflicting results, and our understanding of the modulatory role that the α5 subunit plays remains incomplete. Here, we review these reports and the various techniques used for expression and analysis in order to examine how the α5 subunit modulates key functions in α4β2^*^ and α3β4^*^ receptors, including receptor trafficking, sensitivity, efficacy, and desensitization. In addition, we highlight the strikingly different role that the α5 subunit plays in Ca^2+^ signaling between α4β2^*^ and α3β4^*^ receptors, and we discuss whether the N398 α5 subunit variant can partially replace the D398 variant.

## Introduction

Nicotinic acetylcholine receptors (nAChRs) are homo- and hetero-pentamers that can be distinguished by their sensitivity to α-bungarotoxin. Receptors containing an α4β2^*^^1^ or α3β4^*^ backbone are insensitive to α-bungarotoxin and often referred to as central nervous system (CNS) and peripheral nervous system (PNS) nAChRs, respectively (Millar and Gotti, 2009). Despite this relatively loose distinction, α3β4^*^ receptors have also been found in distinct brain regions such as the medial habenula (MHb) and interpeduncular nucleus (IPN), where they play a central role in nicotine addiction. Moreover, so-called “neuronal” nAChRs are also expressed in non-neuronal cells, where they play both physiological and pathological roles (reviewed by Zoli et al., 2018).

In the PNS, nAChRs mediate synaptic transmission in sympathetic and parasympathetic ganglia. In contrast, nAChRs in the CNS primarily modulate and/or trigger the release of a wide variety of neurotransmitters from presynaptic sites, and the functional impact of this modulation depends on the transmitter system involved (e.g., glutamate, GABA, or catecholamines) and the role these transmitter systems play in brain circuitry.

As ionotropic receptors, nAChRs are ligand-gated cation channels activated by the neurotransmitter acetylcholine (ACh) binding to canonical (orthosteric) binding sites at the N-terminal interface of two subunits with a primary and complementary component (Zoli et al., 2018). The α subunit contains the primary ligand-binding site, and the β subunit contains the complementary site. To maximally activate heteromeric nAChRs, ACh must bind to two binding sites in receptors; in contrast, homomeric α7 receptors require binding to a single site for maximal activation (reviewed by Zoli et al., 2018).

The α3, α4, β2, and β4 subunits can co-assemble to produce a fairly wide range of functional receptor subtypes with various stoichiometries. In the CNS, both high-affinity (α4β2)_2_β2 and low-affinity (α4β2)_2_α4 receptors have been reported (Grady et al., 2010; Marks et al., 2010; Harpsoe et al., 2011). Peripheral-type (α3β4)_2_β4 and (α3β4)_2_α3 receptors have been expressed in *Xenopus* oocytes (Grishin et al., 2010; Krashia et al., 2010; George et al., 2012) and HEK293 cells (Krashia et al., 2010); however, the stoichiometry of α3β4^*^ receptors in the CNS and/or PNS is currently unknown. The fifth subunit—e.g., α4 and β2 in (α4β2)_2_α4 and (α4β2)_2_β2 receptors, and α3 and β4 in (α3β4)_2_α3 and (α3β4)_2_β4 receptors, respectively—may contribute to allosteric binding sites. Receptor complexity is increased further by additional subunits such as the α2, α5, α6, and β3 subunits, which can co-assemble into the two principal types of PNS and/or CNS receptors.

Because both the α5 and β3 subunits lack a primary or complementary component (Le Novere et al., 2002), they cannot contribute to the orthosteric binding site and are therefore often referred to as “accessory” subunits; nevertheless, these subunits contribute to the channel's lining, as well as potential allosteric binding sites and distinct properties conferred by the large cytoplasmic loop between the third and fourth transmembrane domains (i.e., the M3–M4 loop).

Below, we discuss the role of the cytoplasmic loop in the α5 subunit on the receptor's assembly, trafficking, and targeting to the plasma membrane. However, the cytoplasmic loop may also affect other receptor properties beyond its effects on membrane trafficking. For example, Kabbani and colleagues found that the β2 subunit can form a complex with at least 21 different cellular proteins identified using MALDI-TOF-TOF MS/MS analysis (Kabbani et al., 2007).

In addition to their ionotropic properties, nAChRs also mediate G protein signaling via the cytoplasmic loop, as reviewed extensively by Kabbani et al. (2013). The majority of research in this respect has focused on α7-containing nAChRs and found that α7 receptors can act via ionotropic signaling, as well as Gαq-mediated metabotropic signaling via a G protein–binding cluster in the subunit's M3–M4 loop (King et al., 2015). Interestingly, the α3, α5, and β2 subunits have also be been found to bind Goα and Gβγ proteins (Fischer et al., 2005a).

Several putative CaMKII and PKA sites, as well as novel nicotine-induced phosphorylation sites, have been identified in the cytoplasmic loop of α4/β2^*^ nAChRs (Miller et al., 2018). Non-ionic signaling events triggered by nAChR-coupled protein kinases appear to play a particularly prominent role in non-excitable cells, in which receptor activation has been linked to cancer (for review, see Grando, 2014). Although the functional role of α5 phosphorylation has yet to be fully explored, studies involving small molecule kinase inhibitors suggest that kinases do play a functional role related to the α5 subunit (Ray et al., 2017).

The general structure, properties, and function of nAChRs have been covered thoroughly by a large number of reviews (e.g., McGehee and Role, 1995; Le Novere et al., 2002; Gotti et al., 2006; Stokes et al., 2015; Bertrand and Terry, 2018; Zoli et al., 2018). Here, we focus on the α5 accessory subunit, given that human genome-wide association studies have shown that polymorphisms in the gene cluster in chromosomal region 15q25, which includes genes that encode the α5, α3, and β4 nAChR subunits, are linked to susceptibility to nicotine addiction and certain forms of cancer. For example, in the human *CHRNA5* gene, which encodes the α5 nAChR subunit, the single-nucleotide polymorphism (SNP) rs16969968 replaces an aspartic acid with an asparagine in the resulting protein and has been strongly correlated with excessive and compulsive nicotine abuse and lung cancer (see below). On the other hand, the SNP rs16969968 may confer a protective effect against cocaine dependence (Grucza et al., 2008; Forget et al., in press), possibly due to the more general role that the α5 subunit plays in α3β4^*^ and α4β2^*^ receptors, determining whether the function of these receptors is increased or reduced by the presence of an α5 subunit. We will therefore address the role that the α5 subunit plays in α4β2^*^ and α3β4^*^ receptors with respect to their expression, targeting, activation, and desensitization, as well as how the α5 subunit modulates receptor's ability to raise intracellular Ca^2+^.

Considerable insight into the function of the α5 subunit has come from studying recombinant receptors expressed in a wide range of cell types and systems, including *Xenopus* oocytes, HEK293 cells, and rat pituitary GH4C1 cells. In addition, studies using transgenic knockout (KO) mice—and more recently, rats—lacking the α5 subunit have provided new insights into the role of α5 subunits in endogenous receptors. These studies may explain—at least to some extent—the considerable receptor diversity highlighted in Tables 1, 2. However, because the proper assembly and processing of nAChRs in the endoplasmic reticulum is supported by a variety of chaperone proteins, which may not be present in heterologous expression systems, nAChRs should ideally be analyzed in their endogenous physiological context. To date, the properties of native receptors were investigated primarily in mice, whereas the majority of studies involving human nAChRs used heterologous expression systems. As noted above, heterologous expression systems such as the highly popular HEK293 cell line may lack the necessary chaperone proteins such as NACHO required for the assembly and trafficking of nAChRs (Matta et al., 2017). Moreover, heterologous expression systems generally also lack proteins specific to neurons such as the Lynx1 protein (Miwa et al., 1999), which affect the membrane targeting and function of both α3β4^*^ and α4β2^*^ receptors (see below, Nichols et al., 2014; George et al., 2017). Finally, differences in receptor function were found when expressing fully pentameric nAChR concatemer constructs compared to expressing the α3, β4, and α5 subunits in *Xenopus* oocytes (George et al., 2012). Still, some properties of α3β4^*^ receptors differ between human and rodent receptors, as shown by expressing these subunits in *Xenopus* oocytes (Stokes and Papke, 2012). Recently, Maskos reviewed the differences in the properties of receptors containing the N398 α5 subunit variant compared to the more common D398 variant in both α4β2^*^ and α3β4^*^ receptors (Maskos, 2020); we will therefore touch on this subject only briefly. In our review, we focus on the functional effect of the α5 subunit at the cellular level, referring to studies assessing nAChR pathways in nicotine addiction (Leslie et al., 2013; Picciotto and Kenny, 2013; Antolin-Fontes et al., 2015; Pistillo et al., 2015; Molas et al., 2017; Arvin et al., 2019) and variants at the *CHRNA5*/*CHRNA3*/*CHRNB4* gene locus on chromosome 15q25 (Bierut et al., 2008; Stevens et al., 2008; Thorgeirsson et al., 2008; Improgo et al., 2010; Tuesta et al., 2011; Berrettini and Doyle, 2012; Slimak et al., 2014; Lassi et al., 2016; Forget et al., 2018; Besson et al., 2019; Maskos, 2020).

**Table 1:** Effects of α5 in α4β2^*^ receptors.

**Receptor** **without α5** **or with α5^**N398**^**	**Receptor** **with α5^**D398**^**	**Expression system** **or preparation**	**Expression level**	**Potency**	**Efficacy^1^**	**Desensitization acute**	**Desensitization chronic**	**Assay**	**References**
Chick α4β2^*^^2^	(α4β2)_2_α5	*Xenopus*		ACh current ↓^3^	ACh current ↑			Voltage clamp	Ramirez-Latorre et al., 1996
Chick α4β2^*^	(α4β2)_2_α5	*Xenopus*		ACh current ↓	ACh current ↔			Voltage clamp	Fucile et al., 1997
Human (α4β2)_2_β2	(α4β2)_2_α5	*Xenopus*		ACh current ↔	ACh Ca^2+^ permeability ↑			Voltage clamp	Tapia et al., 2007
Human (α4β2)_2_α4	(α4β2)_2_α5	*Xenopus*		ACh current ↑	ACh Ca^2+^ permeability ↑			Voltage clamp	Tapia et al., 2007
Human (α4β2)_2_α5^N398^	(α4β2)_2_α5^D398^	*Xenopus*		ACh current ↔	ACh Ca^2+^ permeability ↑	↓		Voltage clamp	Kuryatov et al., 2011
Human (α4β2)_2_β2	(α4β2)_2_α5	*Xenopus*		ACh current ↔ Saz-A current ↓	ACh current ↓			Voltage clamp	Prevost et al., 2020
Human (α4β2)_2_α4	(α4β2)_2_α5	*Xenopus*		ACh current ↑	ACh current ↓ Saz-A current↑			Voltage clamp	Prevost et al., 2020
Human (α4β2)_2_α5^N398^	(α4β2)_2_α5^D398^	*Xenopus*		ACh current ↔	ACh current ↔	↔		Voltage clamp	Prevost et al., 2020
Mouse (α4β2)_2_β2(α4β2)_2_α4	(α4β2)_2_α5	*Xenopus*		ACh current ↑	ACh current ↓			Voltage clamp	Nichols et al., 2016
Mouse (α4β2)_2_β2	(α4β2)_2_α5	HEK293		ACh ↓				Membrane potential assay kit	Nichols et al., 2016
Human (α4β2)_2_β2	(α4β2)_2_α5	*Xenopus*		ACh current ↔	ACh current ↓			Voltage clamp	Jin et al., 2014
Human (α4β2)_2_α4	(α4β2)_2_α5	*Xenopus*		ACh current ↑	ACh current ↓			Voltage clamp	Jin et al., 2014
Human (α4β2)_2_β2^4^ (α4β2)_2_α4	(α4β2)_2_α5	tsA201	↑ Overall ↓ Cell surface	ACh ↓Nic ↓		↑	↔^5^	Membrane potential and Ca^2+^ assay kits[^3^H]-epibatidine (mAb295, mAb210)	Kuryatov et al., 2008
Mouse^6^ (α4β2)_2_α5^N397^	(α4β2)_2_α5^D397^	HEK293T	↔ Overall	Epi Ca^2+^ ↔	Epi Ca^2+^ ↑			Aequorin [^125^I]-epibatidine	Bierut et al., 2008
α5 KO Mouse	WT Mouse^7^	Thalamus, striatum synaptosomes	↔ Overall	ACh ↔	ACh ↑^8^			^86^Rb^+^ efflux [^125^I]-epibatidine	Brown et al., 2007
α5 KO Mouse	WT Mouse	Thalamus, hindbrain synaptosomes	↔ Overall		ACh ↑^9^			^86^Rb^+^ efflux [^125^I]-epibatidine	Jackson et al., 2010
α5 KO Mouse	WT Mouse	Striatum synaptosomes		ACh ↔	ACh ↑^10^			[^3^H]-DA release	Salminen et al., 2004
α5 KO Mouse	WT Mouse	Dorsal striatum slice			Electrical stimulation ↑^11^			DA release, fast-scan cyclic voltammetry	Exley et al., 2012
α5 KO Mouse	WT Mouse	Prefrontal cortex synaptosomes		ACh ↔	ACh ↑^12^			[^3^H]-GABA release	McClure-Begley et al., 2009
α5 KO Mouse	WT Mouse	Habenula, IPN intact tissue	↔ Overall	Nic ↑				[^3^H]-NE release [^3^H]-epibatidine	Beiranvand et al., 2014
α5 KO Mouse	WT Mouse	Prefrontal cortex synaptosomes					↓^13^	[^3^H]-GABA release	Grady et al., 2012
α5 KO Mouse	WT Mouse	Striatum synaptosomes					↓^14^	[^3^H]-DA release	Wageman et al., 2014
^15^(α4β2)_2_α5^N397^ (α3β4)_2_α5^N397^	WT Mouse	Habenula synaptosomes	↑^16^ Overall	ACh ↔	ACh ↔			^86^Rb^+^ efflux [^125^I]-epibatidine	O'Neill et al., 2018
(α4β2)_2_α5^N397^	WT Mouse	Striatum synaptosomes	↔ Overall		ACh ↑^17^			[^3^H]-DA release [^125^I]-epibatidine	O'Neill et al., 2018
α5 KO Mouse	WT Mouse	Habenula, IPN synaptosomes			ACh ↑^18^			^86^Rb^+^ efflux	Fowler et al., 2011
α5 KO Mouse	WT Mouse	PFC layer VI pyramidal cells, slice		ACh ↑ current	ACh current ↑		↓	Patch clamp	Bailey et al., 2009
α5 KO Mouse	WT Mouse	VTA slice	↑ Overall		ACh current ↑		↓	Patch clamp α4YFP	Chatterjee et al., 2013
α5 KO Mouse	WT Mouse	VTA slice Anesthetized mouse		Firing rate ↑ Nic intravenously	DMPP current ↑			Patch clamp Extracellular recordings	Morel et al., 2014
^19^(α4β2)_2_α5^N397^	WT Mouse	VTA slice Anesthetized mouse		Firing rate ↑ Nic intravenously	DMPP current ↔			Patch clamp Extracellular recordings	Morel et al., 2014
α5 KO Rat	WT Rat	VTA slice Anesthetized rat	↔ Overall	Firing rate ↑ Nic intravenously	DMPP current ↑			Patch clamp Extracellular recordings	Forget et al., 2018
^20^(α4β2)_2_α5^N397^	WT Rat	VTA slice Anesthetized rat	↔ Overall	Firing rate ↔ Nic intravenously	DMPP current ↔			Patch clamp Extracellular recordings	Forget et al., 2018
α5 KO Rat	WT Rat	IPN slice			Nic current ↑			Patch clamp	Forget et al., 2018
(α4β2)_2_α5^N397^	WT Rat	IPN slice			Nic current ↑			Patch clamp	Forget et al., 2018
Human (α4β2)_2_α4	(α4β2)_2_α5^D398^	GH4C1			Nic current ↓↓^21^ Ca^2+^	↔		Patch clamp Fura-2 Ca^2+^ assay	Sciaccaluga et al., 2015
Human (α4β2)_2_α5^N398^	(α4β2)_2_α5^D398^	GH4C1			Nic current ↔↔ Ca^2+^	↓	↑^22^	Patch clamp Fura-2 Ca^2+^ assay	Sciaccaluga et al., 2015
α5 KO Mouse^23^	WT Mouse	Ventral midbrain cell culture			Nic ↑↑^24^ Ca^2+^			Fura-2 Ca^2+^ assay	Sciaccaluga et al., 2015
^25^(α4β2)_2_α5^N397^	WT Mouse	Ventral midbrain cell culture			Nic ↑^26^ Ca^2+^			Fura-2 Ca^2+^ assay	Sciaccaluga et al., 2015
α5 KO Mouse	WT Mouse	Ventral midbrain slice			Nic current ↑^27^			Patch clamp	Sciaccaluga et al., 2015
(α4β2)_2_α5^N397^	WT Mouse	Ventral midbrain slice			Nic current ↑^28^			Patch clamp	Sciaccaluga et al., 2015
(α4β2)_2_α5^N398^	(α4β2)_2_α5^D398^	Dopaminergic iPSC Glutamatergic iPSC		Nic ↓ EPSC frequency			↓^29^	Patch clamp	Oni et al., 2016
α5 KO Mouse	WT Mouse	PFC layer II/III VIP neurons		Firing rate ↑ of VIP interneurons				*In vivo* two-photon calcium imaging	Koukouli et al., 2017
	WT Mouse ^30^(α4β2)_2_α5^N397^	PFC layer II/III VIP neurons		Firing rate ↑ of VIP interneurons				*In vivo* two-photon calcium imaging	Koukouli et al., 2017
α5 KO Mouse	WT Mouse	Rostral IPN slice		Nic current ↑ Nic firing rate ↑	ACh, Nic current ↑			Patch clamp Extracellular recordings	Morton et al., 2018

1 *Deduced from maximal effect at saturating agonist concentration. Unless specifically excluded, an increased efficacy may also result from a higher number of plasma membrane receptors*.

2 *The asterisk means that the two subunits build a backbone, and that an additional subunit will contribute to the fifth position*.

3 *Downward arrow means reduced effect of receptors shown in column 2 (receptor with α5 D398) compared to column 1 (receptor without α5 or with α5 N398)*.

4 *The parent cell line contains a mixture of high-affinity (α4β2)_2_β2 and low-affinity (α4β2)_2_α4 receptors*.

5 *Similar nicotine IC_50_ values*.

6 *In the mouse and rat homologs, amino acid 397 corresponds to amino acid 398 in the human α5 protein*.

7 *WT mice have (α4β2)_2_α5 together with (α4β2)_2_β2 and (α4β2)_2_α4 receptors*.

8 *The α5 KO reduces the DHβE-sensitive component of ^86^Rb^+^ efflux*.

9 *^86^Rb^+^ efflux by 30 μM ACh (high-sensitivity component) is enhanced in WT mice*.

10 *The α-CtxMII–resistant (non-α6) component of dopamine release is reduced in α5 KO mice*.

11 *α4(non-α6) receptors*.

12 *The high-sensitivity component of [^3^H]-GABA release is reduced in α5 KO mice, predominantly in the cortex*.

13 *Higher nicotine IC_50_ values for WT mice*.

14 *(α4β2)_2_β2 (non-α6) are more potently inactivated by nicotine, and recover more slowly from inactivation than (α4β2)_2_α5*.

15 *Mice engineered to express the α5 N397 variant*.

16 *Offsprings were tested: Data show increased cytisine-resistant [^125^I]-epibatidine binding for offsprings from dams with nicotine in drinking water*.

17 *Offsprings were tested. Efficacy for the α-CtxMII resistant component was low for (α4β2)_2_α5^N397^ mice, regardless whether dams had 0.2% saccharin, or nicotine in drinking water; efficacy for α-CtxMII sensitive component was highest in D397 offsprings of dams with 0.2% saccharin in drinking water*.

18 *Injections of Lenti-CHRNA5 into the MHb of knockout mice attenuated the deficits in ^86^Rb efflux in the IPN, but not in the MHb*.

19 *Mice expressing the α5 N397 in the VTA*.

20 *Rats engineered to express the α5 N397 variant*.

21 *Number of cells responding to nicotine; intracellular Ca^2+^ signal likely due to voltage-gated rather than nAChR-mediated Ca^2+^ influx*.

22 *Repetitive application of 100 μM nicotine at one minute intervals with 0.5 mM BAPTA intracellularly*.

23 *Possibly expressing both (α4β2)_2_α4 and (α4β2)_2_β2 receptors*.

24 *None of the α5 KO mouse cells responded to nicotine*.

25 *Mice engineered to possess the α5 N397 variant*.

26 *More WT cells respond to nicotine and also with a higher increase of Ca^2+^*.

27 *No cells with a ≈40 pA (high amplitude) current response in the α5 KO mouse*.

28 *The number of cells with a ≈40 pA current response is significantly reduced in the α5 N397 variant*.

29 *Cells with α5 D398 variant, but not cells with the N398 variant, respond to higher nicotine concentrations by an increase of EPSC frequency*.

30 *Mice engineered to possess the α5 N397 variant*.

*ACh, acetylcholine; DA, dopamine, dopaminergic; Cyt, cytisine; DMPP, dimethylphenylpiperazinium; Epi, epibatidine; EPSC, excitatory postsynaptic current; HEK, human embryonic kidney cells; IPN, interpeduncular nucleus; iPSC, induced pluripotent stem cell; KO, knockout; MHb, medial habenula; NE, norepinephrine; Nic, nicotine; PFC, prefrontal cortex; Saz-A, sazetidine-A; SCG, superior cervical ganglion; Var, varenicline; VIP, vasoactive intestinal polypeptide; VTA ventral tegmental area; WT, wild type*.

**Table 2:** Effects of α5 in α3β4^*^ receptors.

**Receptor without α5 or with α5 N398**	**Receptor with α5 D398**	**Expression system or preparation**	**Expression level**	**Potency**	**Efficacy^1^**	**Desensitization acute**	**Desensitization chronic**	**Assay**	**Reference**
Human α3β4^*^^2^	(α3β4)_2_α5	*Xenopus*	Cell surface ↔	ACh, Nic ↔	ACh, Nic current ↔	↑^3^		[^125^I]-mAb210 Voltage clamp	Wang et al., 1996
Chick α3β4^*^	(α3β4)_2_α5	*Xenopus*		ACh ↔	ACh current ↔			Voltage clamp	Fucile et al., 1997
Chick α3β4^*^	(α3β4)_2_α5	BOSC-23		ACh ↓^4^	ACh current ↓	↔		Patch clamp	Fucile et al., 1997
Human α3β4^*^	(α3β4)_2_α5	*Xenopus*		ACh, DMPP, Cyt ↔	DMPP current ↓ Ca^2+^ permeability ↑	↑		Voltage clamp	Gerzanich et al., 1998
Human (α3β4)_2_α5^N398^	(α3β4)_2_α5^D398^	*Xenopus*		ACh ↔	ACh Ca^2+^ permeability ↔	↔		Voltage clamp	Kuryatov et al., 2011
Human α3β4^*^	(α3β4)_2_α5	*Xenopus*		ACh ↔	ACh current ↔	↑		Voltage clamp	Groot-Kormelink et al., 2001
Mouse α3β4^*^	(α3β4)_2_α5	*Xenopus*		ACh ↔^5^				Voltage clamp	Papke et al., 2010
Human (α3β4)_2_β4	(α3β4)_2_α5	*Xenopus*		ACh, Nic, Cyt, Var ↔^6^				Voltage clamp	Stokes and Papke, 2012
Human (α3β4)_2_α5^N398^	(α3β4)_2_α5^D398^	*Xenopus*		ACh, Nic, Cyt, Var ↔				Voltage clamp	Stokes and Papke, 2012
Human (α3β4)_2_β4	(α3β4)_2_α5	*Xenopus*		ACh, Nic, Cyt ↔	ACh, Nic, Cyt current ↑			Voltage clamp	George et al., 2012
Human (α3β4)_2_α3	(α3β4)_2_α5	*Xenopus*		ACh, Nic, Cyt ↔	ACh, Nic, Cyt current ↑			Voltage clamp	George et al., 2012
Human (α3β4)_2_α5^N398^	(α3β4)_2_α5^D398^	*Xenopus*		ACh, Nic, Cyt ↔	ACh, Nic, Cyt current ↔↑^7^			Voltage clamp	George et al., 2012
Mouse (α3β4)_2_β4	(α3β4)_2_α5	*Xenopus*			ACh current ↓^8^			Voltage clamp	Frahm et al., 2011
Mouse (α3β4)_2_α5^N397^	(α3β4)_2_α5^D397^	*Xenopus*			ACh current ↑^9^			Voltage clamp	Frahm et al., 2011
Human α3β4^*^	(α3β4)_2_α5^10^	tsA201	Overall ↔	ACh, Nic ↔		↔		[^3^H]-epibatidine Patch clamp	Wang et al., 1998
Human α3β4^*^	(α3β4)_2_α5^11^	tsA201		ACh, Nic, Cyt, DMPP ↔		ACh ↔		Patch clamp	Nelson et al., 2001
Human α3β4^*^	^12^(α3β4)_2_α5^D398^	HEK293		ACh, Nic, Cyt, DMPP ↔		↔↓^13^	↔^14^	Patch clamp	Li et al., 2011
Human (α3β4)_2_α5^N398^	(α3β4)_2_α5^D398^	HEK293		ACh, Nic, Cyt, DMPP ↔		↔	↔^15^	Patch clamp	Li et al., 2011
Human α3β4^*^	(α3β4)_2_α5	HEK293	Overall ↔ Cell surface ↔	Nic, ACh, Var ↔	Nic, Var Ca^2+^ ↓	↔^16^	↔	mAb35 [^125^I]-epibatidine Aequorin Ca^2+^ assay	Tammimaki et al., 2012
Human (α3β4)_2_α5^N398^	(α3β4)_2_α5^D398^	HEK293	Overall ↔ Cell surface ↔	Nic ↑^17^ ACh, Var ↔	Nic, ACh, Var Ca^2+^ ↔	↔^18^	↔	mAb35 [^125^I]-epibatidine Patch clamp Aequorin Ca^2+^ assay	Tammimaki et al., 2012
Human α3β4^*^	(α3β4)_2_α5	HEK293	Cell surface ↓		Nic Ca^2+^ ↓		↓	Tagged subunits Aequorin Ca^2+^ assay	Ray et al., 2017
Human (α3β4)_2_α5^N398^	(α3β4)_2_α5^D398^	HEK293			Nic Ca^2+^ ↑			Tagged subunits Aequorin Ca^2+^ assay	Ray et al., 2017
Human (α3β4)_2_α5^N398^	(α3β4)_2_α5^D398^	DA iPSC		ACh, Nic ↑	ACh, Nic current ↓	↔		Patch clamp	Deflorio et al., 2016
Human α3β4^*^	(α3β4)_2_α5	Rat kidney cells	Cell surface ↓					Confocal microscopy	Crespi et al., 2018b
α5 KO Mouse	WT mouse	Habenula intact tissue	Overall ↔					[^3^H]-epibatidine	Scholze et al., 2012
Chick ^19^α3β4^*^	Chick (α3β4)_2_α5	Sympathetic neurons			ACh, Cyt ↓			Patch clamp	Yu and Role, 1998
α5β2 KO Mouse^20^	β2 KO Mouse^21^	SCG cell culture	Overall ↔	Cyt, DMPP current ↔	ACh, Cyt, DMPP current ↔	↔		[^3^H]-epibatidine Patch clamp	David et al., 2010
α5 KO Mouse	WT mouse	SCG cell culture		ACh, Nic, Cyt, DMPP, Epi ↔	ACh, Nic, Cyt, DMPP, Epi ↓			[^3^H]-NE release Fura-2 Ca^2+^ assay	Fischer et al., 2005b
α5 KO Mouse	WT mouse	SCG intact ganglion		↔^22^	↔		↓	Transganglionic transmission	Simeone et al., 2019

1 *Deduced from maximal effect at saturating agonist concentration. Unless specifically excluded, the increased efficacy may also result from a higher number of plasma membrane receptors*.

2 *The asterisk means that the two subunits build a backbone, and that an additional subunit will contribute to the fifth position*.

3 *Upward arrow means enhanced effect of receptors shown in column 2 (receptor with α5 D398) compared to column 1 (receptor without α5 or with α5 N398)*.

4 *Co-expression of α5 leads to a biphasic concentration-response curve due to the appearance of a second low-affinity component*.

5 By comparison of EC_50_ values of peak currents; the α3β4^*^ concentration-response curve for net charge is biphasic.

6 *Comparison of EC_50_ values of peak currents*.

7 *Significantly different for ACh; enhanced but not significantly different for nicotine and cytisine in WT*.

8 *With ratios of 10:10:1 for α5:β4:α3 injected cRNA, α5 will reduce currents compared to oocytes injected with β4:α3 at a ratio of 10:1*.

9 *With ratios of 10:10:1 for α5:β4:α3 injected cRNA, currents by α5 D397 are larger than currents by α5 N397*.

10 Only 14% of α3β4^*^ receptors contain the α5 subunit.

11 Only 14% of α3β4^*^ receptors contain the α5 subunit.

12 *A FLAG epitope was inserted near the amino terminus of the α5 subunit. Cells were selected by binding to beads coated with antibody to the FLAG epitope*.

13 *Decay time not significantly different for 1 mM ACh; significantly prolonged for 100 μM nicotine*.

14 *Recovery from desensitization*.

15 *Recovery from desensitization*.

16 *Residual current after a 40 s pulse of 100 μM ACh, recorded by patch clamp electrophysiology (bath solution with 2 mM Ca^2+^)*.

17 *(α3β4)_2_α5^D398^ is significantly more sensitive than (α3β4)_2_α5^N398^*.

18 *Residual current after a 40 s pulse of 100 μM ACh, recorded by patch clamp electrophysiology (bath solution with 2 mM Ca^2+^)*.

19 *AS: antisense oligonucleotide treatment*.

20 *Remaining receptors are 100% α3β4*.

21 *Remaining receptors are 75% α3β4 and 25% (α3β4)_2_α5*.

22 *Unaltered amplitude of compound action potential and EPSC*.

*ACh, acetylcholine; DA, dopamine, dopaminergic; Cyt, cytisine; DMPP, dimethylphenylpiperazinium; Epi, epibatidine; EPSC, excitatory postsynaptic current; HEK, human embryonic kidney cells; IPN, interpeduncular nucleus; iPSC, induced pluripotent stem cell; KO, knockout; MHb, medial habenula; NE, norepinephrine; Nic, nicotine; PFC, prefrontal cortex; Saz-A, sazetidine-A; SCG, superior cervical ganglion; Var, varenicline; VIP, vasoactive intestinal polypeptide; VTA ventral tegmental area; WT, wild type*.

**Figure 1:**
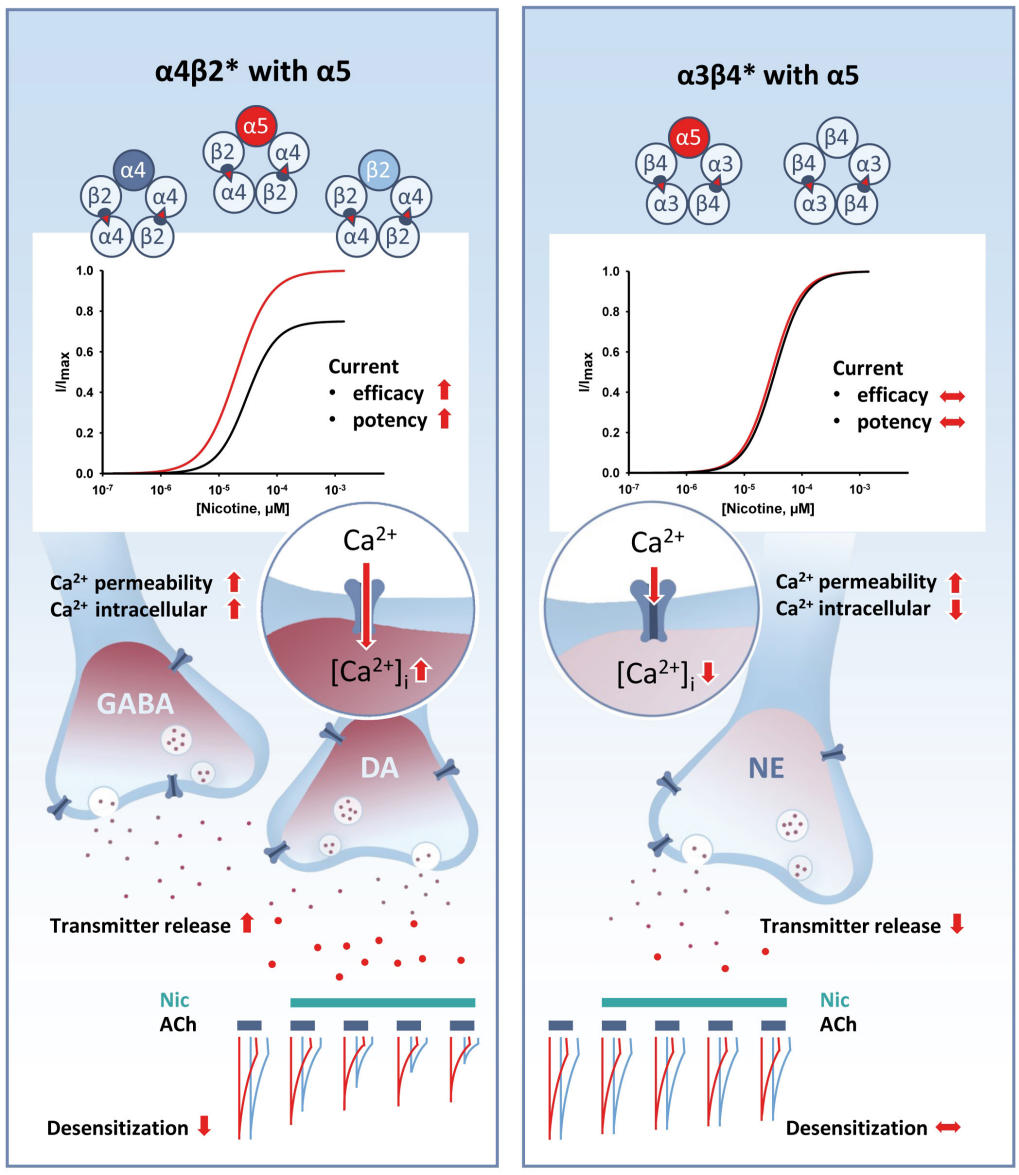
Graphical summary of the key effects of the α5 subunit on various receptor properties when co-assembled with either α4β2* or α3β4* receptors. Red arrows indicate effects mediated by the presence of α5. Left: addition of the α5 subunit to α4β2* receptors increases ligand efficacy and potency (top), increases Ca^2+^ permeability and transmitter release (middle), and decreases receptor desensitization (bottom). Right: in contrast, addition of the α5 subunit to α3β4* receptors has no effect on efficacy or potency (top), increases Ca^2+^ permeability while decreasing intracellular Ca^2+^ and transmitter release (middle), and has no significant effect on receptor desensitization (bottom). Note: that in cases in which the reported effects of the α5 subunit differed between exogenously expressed receptors and endogenous receptors, we report the results observed for endogenous receptors.

The vast majority of heteropentameric neuronal nAChRs consist of two α subunit and two β subunits that comprise the backbone, with an additional subunit completing the pentamer (reviewed in Zoli et al., 2018). This additional subunit can be an “accessory” subunit such as α5 or β3, or it can be a “complementary” subunit such as α4 or β2 (primarily in CNS-type receptors), or α3 or β4 (primarily in PNS-type receptors). The two receptor backbones into which the α5 subunit can co-assemble, namely α4β2^*^ (see Table 1) and α3β4^*^ (see Table 2), differ fundamentally with respect to both their activation and desensitization properties and are discussed separately. These differences in receptor properties have consequences with respect to tobacco use, as nicotine concentrations typically reached while smoking tobacco primarily activate—and equally important, inactivate—α4β2^*^ receptors (Benowitz and Jacob, 1990; Wooltorton et al., 2003; Brody et al., 2006). It is therefore interesting to examine how the α5 subunit affects the properties of these two receptor subtypes, and how the D398 and N398 α5 subunit variants differ in this respect. A graphical summary of the effects of α5 is provided in Figure 1.

## What We Already Know From Heterologously Expressed and Endogenous Receptors

### The α5 Subunit Co-assembles With α4 and β2 in the Central Nervous System

The presence of the α5 subunit may affect the functional properties of nAChRs in several ways, including: (i) altering the potency and efficacy of ligands; (ii) affecting the receptor's Ca^2+^ permeability (or altering other mechanisms that increase intracellular Ca^2+^); (iii) altering the receptor's desensitization properties; (iv) regulating receptor expression, posttranslational processing, and/or trafficking to the cell membrane; and (v) modulating Ca^2+^-independent downstream signaling. Moreover, the anatomical distribution of α5 affects the functional role of nAChRs in the CNS.

#### Distribution of α5-Containing Receptors in the Central Nervous System

Our knowledge regarding the distribution of α5-containing receptors is based on *in situ* hybridization (Wada et al., 1990; Azam et al., 2002; Winzer-Serhan and Leslie, 2005), antibody-based techniques such as immunoprecipitation (Mao et al., 2006; Grady et al., 2009; David et al., 2010) and solid-phase radioimmunoassay (Conroy and Berg, 1995; Wang et al., 1998), expressing reporter genes under the control of the *CHRNA5* promoter (Hsu et al., 2013; Morton et al., 2018), and electrophysiology and optical recordings in specific regions of the nervous system (e.g., Morel et al., 2014). Unfortunately, reliable anti-α5 antibodies for use in immunocytochemistry are not currently available.

A recent RNAseq study revealed that 9 out of 16 nAChR subunits genes (among them most notably *CHRNA4* and *CHRNB2*, but also at moderate levels *CHRNA5*) are already expressed early in human brain development between 7.5 and 12 post-conceptional weeks (Alzu'bi et al., 2020). In adult rodent brain, immunoprecipitation experiments using α5-specific antibodies have shown the presence of α5-containing receptors—but not an association between α5 subunits and α4 and/or β2 subunits—in certain regions in the rodent brain, including the MHb, IPN, hippocampus, striatum, thalamus, prefrontal cortex (PFC), substantia nigra, ventral tegmental area (VTA), brainstem, and spinal cord (Brown et al., 2007; Counotte et al., 2012; Xanthos et al., 2015; Forget et al., 2018). In addition, measuring α5 subunits in mice lacking specific nAChR subunits (e.g., α4 or β2) has provided evidence that α5 associates with the α4 and β2 subunits (Champtiaux et al., 2003; Grady et al., 2009; Scholze et al., 2012; Beiranvand et al., 2014; Xanthos et al., 2015). The direct association between α5 and the α4β2^*^ backbone was demonstrated using a combination of immunopurification and immunoprecipitation of [^3^H]-epibatidine–labeled receptors with subunit-specific antibodies. Studies in the rat CNS indicate that (α4β2)_2_α5 receptors are expressed robustly in several regions, including the hippocampus, striatum, cerebral cortex, thalamus, and superior colliculus (Zoli et al., 2002; Mao et al., 2008; Grady et al., 2009). Similar experiments in mice revealed that these receptors are expressed in the striatum (Champtiaux et al., 2003), superior colliculus, and lateral geniculate nucleus (Gotti et al., 2005). Finally, sequential immunoprecipitation experiments revealed the expression of (α4β2)_2_α5 receptors in the chick brain (Conroy and Berg, 1998) and human neocortex (Gerzanich et al., 1998).

Two brain regions in which α5-containing receptors are expressed have been shown to play a key role in the reinforcing effects of nicotine; these regions are the MHb-IPN system, which accounts primarily for withdrawal mechanisms, and the VTA, which principal role consists in mediating reward (Tuesta et al., 2011; Leslie et al., 2013; Picciotto and Kenny, 2013; Antolin-Fontes et al., 2015; Pistillo et al., 2015; Molas et al., 2017; Arvin et al., 2019). Additional evidence suggests that—irrespective of the α5 subunit—α4β2 and α6β2 receptors in the VTA are necessary and sufficient for systemic nicotine reinforcement (Pons et al., 2008). Finally, Champtiaux and colleagues reported that the α5 subunit preferentially associates with the α4 and β2 subunits in dopaminergic neurons (Champtiaux et al., 2003).

Interestingly, α5-containing receptors in the MHb-IPN system have been linked to tobacco abuse and thus warrant special attention. No other region in the CNS has such a high density of nAChRs, and no region expresses more α5-containing receptors than the IPN. For example, the IPN of adolescent or adult rat contains ~350 fmol of overall receptor protein/mg total protein (Grady et al., 2009; Forget et al., 2018), and even though the reported amount of α5-containing receptors differs between studies, ranging from 23 fmol/mg protein (Forget et al., 2018) to 200 fmol/mg protein (Grady et al., 2009), direct comparisons between various brain regions support the notion that α5-containing receptors are highly enriched in both the rat (Forget et al., 2018) and mouse IPN (Beiranvand et al., 2014; Xanthos et al., 2015). Immunodepletion using an anti-β2 antibody significantly reduced the number of α5-containing receptors in the rat IPN, suggesting that the α5 subunit co-assembles into β2-containing receptors (Grady et al., 2009). In contrast, α5-containing receptors are not reduced in the IPN of β2 KO mice (Grady et al., 2009), although a different study found that α5-containing receptors were significantly reduced in β2 KO mice, but not in β4 KO mice (Beiranvand et al., 2014). Given that the levels of α4—but not α3—subunits are significantly reduced in both β2 KO mice (Grady et al., 2009; Beiranvand et al., 2014) and β2-immunodepleted rats (Grady et al., 2009), we conclude that α5 predominantly, if not exclusively, assembles into α4β2^*^ receptors in the rodent IPN. As summarized below, a strikingly different picture emerges with respect to the MHb, in which the α5 subunit serves as the accessory subunit in α3β4^*^ receptors (Scholze et al., 2012).

Using a transgenic α5^GFP^ mouse, Hsu and colleagues found that α5-containing receptors are robustly expressed in several IPN subnuclei, but not in the MHb; the α5-containing neurons in the IPN were identified as predominantly GABAergic neurons that project to distinct raphe nuclei (Hsu et al., 2013). In a follow-up study by the same group, these findings were confirmed and expanded using *Chrna5*^Cre^ mice with the *Ai6* reporter gene (Morton et al., 2018). Specifically, they performed electrophysiological recordings in acute brain slices and found that the α5 subunit co-assembles with α4 and β2 subunits in these neurons, and currents induced by applying 1 mM ACh were significantly reduced by 10 μM dihydro-β-erythroidine (DhβE), which preferentially inhibits α4β2^*^ receptors at this concentration (Morton et al., 2018).

Functional evidence supporting the presence of α5-containing nAChRs in distinct cell types in the CNS comes from both *in vivo* and *in vitro* (e.g., brain slices, transmitter release, etc.) experiments. For example, experiments combining patch-clamp recordings with single-cell PCR found that the nicotine-induced activation of interneurons is mediated by nAChRs composed of α4, α5, and β2 subunits in layers II, III, and V in acute rat brain slices containing the motor neocortex (Porter et al., 1999). Moreover, nAChR agonists induced larger, DhβE-sensitive currents in layer VI pyramidal neurons in slices containing the medial PFC taken from mice expressing the α5 subunit compared to slices obtained from α5 KO mice (Bailey et al., 2009). More recently, Koukouli and colleagues performed two-photon Ca^2+^ imaging in awake α5 KO mice and found reduced activity of VIP (vasoactive intestinal polypeptide)–expressing GABAergic interneurons, affecting the firing rate in layer II/III pyramidal cells (Koukouli et al., 2017). Finally, the α5 subunit has been shown to play a critical role in midbrain VTA neurons, in which the presence of this subunit significantly increased both the overall number of α4-containing receptors and the magnitude of ACh-induced, DhβE-sensitive currents (Chatterjee et al., 2013).

In summary, the α5 subunit co-assembles with the α4 and β2 subunits in different regions throughout the CNS; the medial habenula also expresses relatively low levels of (α3β4)_2_α5 nAChRs.

#### Receptor Affinity and Efficacy

The functional properties of α4β2^*^ receptors, and how these properties are affected by the presence of the α5 accessory subunit, have been studied in detail using heterologous expression systems. For example, seminal work by Ramirez-Latorre and colleagues showed that chick α5 subunits require both the α4 and β2 subunits to form functional receptors when expressed in *Xenopus* oocytes, and the concentration-response curve of ACh-induced currents in α4β2 receptors was significantly right-shifted, with larger current amplitude, when the α5 subunit was expressed (Ramirez-Latorre et al., 1996). These early observations were confirmed partially by individual constructs or the pairwise expression of human α4β2 concatemers together with α4, β2, or α5 subunits in *Xenopus* oocytes; specifically (α4β2)_2_α5 receptors were as sensitive to ACh as (α4β2)_2_β2 receptors, while the concentration-response curve was significantly right-shifted for (α4β2)_2_α4 receptors compared to (α4β2)_2_β2 receptors (Tapia et al., 2007; Jin et al., 2014). More recently, Nichols and colleagues injected *Xenopus* oocytes with α5, α4, and β2 mRNA at a 10:1:1 ratio (i.e., a 10-fold excess of α5) and found that 100% of the receptors were high-affinity (i.e., α5 subunit-containing), with an ACh EC_50_ of 0.26 μM); in contrast, oocytes injected with only α4 and β2 (at a 1:1 ratio) had a biphasic concentration-response, with 65% high-affinity receptors (ACh EC_50_: 0.67 μM) and 35% low-affinity receptors (ACh EC_50_: 190 μM) (Nichols et al., 2016).

Lately, Prevost and colleagues used pentameric concatemer constructs for expression in *Xenopus* oocytes (Prevost et al., 2020). Similar to previous reports they observed no difference in ACh potency between human (α4β2)_2_β2 and (α4β2)_2_α5 receptors, whereas (α4β2)_2_α4 receptors showed a biphasic concentration response curve with an overall significantly reduced ACh potency. However, current amplitudes in response to saturating ACh concentrations were significantly reduced for α5 containing concatemers. Sazetidine-A, on the other hand, was a partial agonist for (α4β2)_2_α4 but a full agonist for (α4β2)_2_β2 and (α4β2)_2_α5 receptors, albeit with lower potency for (α4β2)_2_α5 receptors. Of interest, α5-containing nAChRs were irreversibly blocked by methanethiosulfonate reagents through a covalent reaction with a cysteine present at the second transmembrane segment only in α5 at position 261. By using this approach, the authors showed that reconstitution of nAChRs from loose α5, α4 and β2 subunits was inefficient and highly variable (Prevost et al., 2020).

Importantly, the presence of the α5 subunit also significantly increases the receptor's Ca^2+^ permeability. With Ca^2+^ as the only cation available in the superfusion buffer, peak currents recorded in (α4β2)_2_α5–expressing *Xenopus* oocytes were even larger than currents measured in oocytes expressing α7 receptors, the nAChR subtype with the highest Ca^2+^ permeability; (α4β2)_2_α4 receptors also showed high Ca^2+^ permeability, whereas (α4β2)_2_β2 receptors were hardly Ca^2+^-permeable (Tapia et al., 2007). Using stable nAChR-expressing tsA201 cell lines, Kuryatov and colleagues found that (α4β2)_2_β2 receptors were most sensitive to nicotine and ACh, followed by (α4β2)_2_α5, and then (α4β2)_2_α4, similar to the previously reported overall ranking measured in *Xenopus* oocytes (Kuryatov et al., 2008). Moreover, immunoisolation experiments revealed that 100% of receptors in the (α4β2)_2_α5-expressing cell line indeed contained the α5 subunit (Kuryatov et al., 2008).

Any increase in Ca^2+^ permeability will affect downstream Ca^2+^-dependent processes such as nAChR-induced transmitter release. Consistent with this notion, ACh-induced, DhβE-sensitive ^86^Rb efflux was significantly smaller in thalamic synaptosomes prepared from α5 KO mice compared to wild-type (WT) mice^2^. The finding that [^125^I]-epibatidine binding was not affected in the α5 KO mice—indicating that the overall number of receptors is unchanged—suggests impaired function in α4β2^*^ receptors that lack the α5 accessory subunit (Brown et al., 2007; Jackson et al., 2010). Although ^86^Rb efflux measures the overall function of synaptic release, independent of the underlying transmitter system, these results are supported by the finding that α-conotoxin MII (α-CtxMII)–resistant, DhβE-sensitive [^3^H]-dopamine efflux from mouse striatal synaptosomes was also reduced in the α5 KO mouse (Salminen et al., 2004). These observations were subsequently confirmed by showing that dopamine release (measured using fast-scan cyclic voltammetry) requires the α5 subunit in the dorsal striatum, but not in the nucleus accumbens (Exley et al., 2012). Finally, the high-affinity component of ACh-induced GABA release from synaptic vesicles, which was abolished in several brain regions in α4 and β2 KO mice, was significantly reduced in the PFC—as well as in the hippocampus and striatum, albeit to a lesser extent—upon deletion of the α5 subunit (McClure-Begley et al., 2009).

In both the MHb and IPN, the nicotine concentration-response curves for the release of norepinephrine were right-shifted in α5 KO mice compared to control mice, suggesting that the α5 subunit increases the receptor's ligand sensitivity (Beiranvand et al., 2014). However, nAChR-stimulated norepinephrine release requires action potentials (Sacaan et al., 1995; Scholze et al., 2007) and is blocked by tetrodotoxin (TTX), similar to nicotine-induced norepinephrine release in the hippocampus (Sacaan et al., 1995; Scholze et al., 2007; Beiranvand et al., 2014). Moreover, the Ca^2+^ required for synaptic vesicle fusion (and hence, transmitter release) in presynaptic terminals may come either via nACh receptors (if they are positioned closely enough to the release site) or via voltage-gated Ca^2+^ channels (along with the action potentials generated by nAChRs); these two mechanisms have been termed “transmitter release by presynaptic nAChRs receptors” and “transmitter release by preterminal nAChRs receptors,” respectively (Wonnacott, 1997). In addition, norepinephrine release was also abolished in β2 KO mice, suggesting that this release is mediated by α4β2^*^ receptors (Scholze et al., 2007; Beiranvand et al., 2014). Importantly, both the MHb (Lecourtier and Kelly, 2007) and the IPN (Antolin-Fontes et al., 2015) receive noradrenergic input from the locus coeruleus, where most nicotinic subunits, including α5, are expressed (Lena et al., 1999).

Patch-clamp recordings revealed significantly smaller currents in response to 1 mM ACh in brain slices containing the VTA prepared from α5 KO mice compared to WT mice (Chatterjee et al., 2013). Likewise, stimulating nAChRs in VTA brain slices with a saturating concentration of dimethylphenylpiperazinium (DMPP) induced smaller currents in α5 KO mice compared to WT mice, and restoring the α5 subunit in α5 KO mice by lentiviral infection restored the DMPP-induced response in VTA neurons to WT levels (Morel et al., 2014). Consistent with these results, an 8-fold higher dose of intravenous nicotine was required to significantly increase the *in vivo* firing rate of dopaminergic VTA neurons in α5 KO mice compared to WT mice, and the sensitivity to intravenous nicotine was restored by expressing the α5 subunit in α5 KO VTA dopaminergic neurons (Morel et al., 2014). These observations in mice were later supported in a follow-up study using α5 KO rats, in which currents induced with 100 μM DMPP were significantly smaller in VTA brain slices prepared from KO rats compared to WT rats (Forget et al., 2018). Similarly, a 3-fold higher intravenous dose of nicotine was needed to increase the firing frequency of VTA neurons in KO rats compared to WT rats. Consistent with affecting function but not expression, the overall number of nAChRs in nine specific brain regions (measured using immunoprecipitation) was similar between WT and KO animals (Forget et al., 2018). Finally, currents elicited with 30 μM nicotine were significantly smaller in IPN slices prepared from α5 KO rats compared to WT rats (Forget et al., 2018). Taken together, these findings suggest that the α5 accessory subunit increases the receptor's sensitivity and efficacy.

Interestingly, cells recorded in VTA slices prepared from α5 KO mice lacked an additional “large” (40 pA) current component measured in response to 100 μM nicotine, a feature that was present in WT slices (Sciaccaluga et al., 2015). In addition, the authors also found that applying 100 μM nicotine increased intracellular Ca^2+^ in cultured VTA neurons prepared from WT mice, but not in neurons prepared from α5 KO mice (Sciaccaluga et al., 2015). Overall, these findings suggest that the α5 subunit increases the receptor's efficacy.

On the other hand, Sciaccaluga and colleagues found that expressing the α5 subunit in rat pituitary GH4C1 cells significantly reduced receptor efficacy compared to cells expressing (α4β2)_2_α4 receptors. Hence, cells expressing (α4β2)_2_α5 receptors had smaller currents and less increase in intracellular Ca^2+^ in response to 100 μM nicotine (Sciaccaluga et al., 2015). This finding is consistent with reduced receptor efficacy (measured using voltage-clamp recordings) in *Xenopus* oocytes expressing (α4β2)_2_α5 receptors compared to (α4β2)_2_α4 receptors (Jin et al., 2014). Nevertheless, given the increased Ca^2+^ permeability of (α4β2)_2_α5 receptors (Tapia et al., 2007), one would have expected increased Ca^2+^ signals in GH4C1 cells expressing these receptors.

The α5 subunit is also expressed in VIP interneurons in layer II/III, which inhibit both somatostatin and parvalbumin interneurons in the PFC. Given that both somatostatin and parvalbumin GABAergic neurons reduce the firing rate of layer II/III pyramidal neurons, reduced activity of VIP interneurons would be expected to reduce the firing frequency of pyramidal neurons. Consistent with this hypothesis, Koukouli and colleagues performed *in vivo* two-photon Ca^2+^ imaging in awake mice and found that pyramidal cells in α5 KO mice fire at a reduced frequency, and targeted virus-mediated expression of the α5 subunit in VIP GABAergic neurons restored both the normal firing rate of VIP interneurons and the firing frequency of pyramidal cells (Koukouli et al., 2017). Together with the *in vitro* data above, these *in vivo* results suggest that (α4β2)_2_α5 receptors have a robust response to endogenous ACh compared to receptors lacking the α5 subunit.

The presence of the α5 subunit in α4β2^*^ receptors also appears to have a major effect on neuronal activity in the rostral IPN, as currents elicited in response to 1 μM nicotine or 1 mM ACh are smaller in slices prepared from α5 KO mice compared to WT mice; in contrast, no difference was observed in the ventral MHb (Morton et al., 2018). The reduced response in α5 KO IPN neurons could be due to a reduced number of receptors and/or reduced efficacy; based on previously published [^3^H]-epibatidine radioligand binding experiments in WT and α5 KO mice, the authors concluded that reduced efficacy of receptors lacking the α5 subunit is the more likely explanation (Morton et al., 2018). Thus, the reduced DhβE-sensitive ACh-induced currents in layer VI pyramidal neurons in α5 KO mice are likely also due to reduced efficacy, rather than reduced receptor expression or membrane trafficking (Bailey et al., 2009). Finally, Bailey and colleagues found that ACh is not only less efficacious but also less potent in eliciting currents in layer VI pyramidal neurons in α5 KO mice compared to WT mice (Bailey et al., 2009).

In summary (α4β2)_2_α5 and (α4β2)_2_β2 receptors have similar sensitivity, and both receptor subtypes are significantly more sensitive than (α4β2)_2_α4 receptors, which are also found in the CNS. Moreover, agonists are more efficacious at activating (α4β2)_2_α5 receptors compared to α4β2^*^ receptors lacking the α5 subunit, while (α4β2)_2_α5 receptors have higher Ca^2+^ permeability compared to both (α4β2)_2_β2 and (α4β2)_2_α4 receptors. Taken together, these properties explain the fact that (α4β2)_2_α5 receptors are highly efficacious at mediating transmitter release in the CNS.

#### Desensitization Properties

Unlike acute receptor desensitization, seen as the decay of current during ligand application, “prolonged” desensitization may actually be more physiologically relevant. Receptors enter and maintain a state of prolonged desensitization when exposed to ligands such as nicotine (at concentrations measured in smokers) for an extended period of time (Quick and Lester, 2002; Wooltorton et al., 2003).

The effect of the α5 subunit on prolonged desensitization in nAChRs has been studied in both heterologously expressed and endogenous receptors, with partially conflicting results. When expressed in tsA201 cells, for example, long-term (e.g., 6 h) desensitization was similar between cells expressing (α4β2)_2_α5 and cells expressing a combination of (α4β2)_2_α4 and (α4β2)_2_β2 receptors (Kuryatov et al., 2008). In contrast, several reports found that the α5 subunit reduces desensitization when assembled in α4β2^*^ receptors. For example (α4β2)_2_β2 receptors mediating GABA release in cortical synaptosomes isolated from α5 KO mice had significantly more nicotine-induced desensitization compared to the (α4β2)_2_α5 receptors present in WT synaptosomes (Grady et al., 2012). Similarly, the IC_50_ values of 11 agonists for desensitizing α-CtxMII-resistant (i.e., non-α6^*^) receptors that mediate [^3^H]-dopamine release were significantly lower in α5 KO striatal synaptosomes compared to WT (Wageman et al., 2014).

By measuring currents induced by applying 1 mM ACh to layer VI pyramidal cells in the medial PFC, Bailey and colleagues found that neurons in α5 KO mice had ~2-fold more desensitization following a 10-min pretreatment with 100 or 300 nM nicotine compared to WT neurons (Bailey et al., 2010). In extending this observation by optogenetic stimulation of cholinergic fibers, Venkatesan and Lambe recently reported that in α5 WT mice, the optogenetic cholinergic response of layer VI pyramidal cells is unchanged by application of 100 nM nicotine, whereas the optogenetic response is rapidly attenuated in α5 KO mice (Venkatesan and Lambe, 2020). Interesting, 300 nM nicotine cause complete desensitization of receptors in layer II/III and layer VI interneurons measured using patch-clamp recordings in PFC slice preparations; in contrast, the cholinergic responses in layer V interneurons and layer VI pyramidal cells had less desensitization, possibly due to the expression of the α5 subunit in these neurons (Poorthuis et al., 2013). Finally, Chatterjee and colleagues found significantly more nicotine-induced desensitization in VTA neurons in slices prepared from α5 KO compared to WT mice (Chatterjee et al., 2013).

In summary, α5 KO neurons are more sensitive to chronic agonist-induced desensitization compared to WT neurons.

#### Receptor Expression and Membrane Trafficking

Using tsA201 cell lines expressing either α4β2^*^ or (α4β2)_2_α5 receptors, Kuryatov and colleagues found that cells expressing (α4β2)_2_α5 receptors had 40% more epibatidine binding sites compared to cells expressing α4β2^*^ receptors [i.e., (α4β2)_2_α4 and (α4β2)_2_β2 receptors], suggesting that the presence of the α5 subunit increases overall receptor expression; in contrast, plasmic membrane targeting [which in cells expressing (α4β2)_2_α5 receptors represents ~20% of the total [^3^H]-epibatidine–binding pool] was significantly reduced compared to cells expressing α4β2^*^ receptors (Kuryatov et al., 2008). The α5 subunit has been shown to play a role in the expression of α4-containing receptors midbrain VTA neurons, increasing the overall expression and trafficking of α4β2^*^ receptors (Chatterjee et al., 2013). In contrast, [^125^I]-epibatidine binding measured at a high enough concentration to bind both high-affinity and low-affinity receptors was similar between WT and α5 KO mice in all brain regions investigated (Salas et al., 2003). A subsequent study by Baddick and Marks using semi-quantitative visual analysis in WT and additional mouse KO models confirmed these results (Baddick and Marks, 2011). As discussed above, Brown and colleagues found that deleting the α5 subunit in α5 KO mice significantly reduced DhβE-sensitive ^86^Rb efflux in thalamic synaptosomes without affecting [^125^I]-epibatidine binding, suggesting reduced receptor efficacy, rather than reduced expression of presynaptic receptors (Brown et al., 2007). Interestingly, Nichols and colleagues found that introducing the V287L mutation in the β2 subunit (a mutation linked to autosomal dominant nocturnal frontal lobe epilepsy) reduced the total surface expression of α4β2^*^ receptors expressed in HEK293 cells but caused a 4-fold increase in (α4β2)_2_α5 receptors at the plasma membrane (Nichols et al., 2016).

In summary, in both mice and rats, loss of α5 does not affect the overall expression of α4β2^*^ receptors measured using either [^125^I]-epibatidine or [^3^H]-epibatidine binding.

#### Do the Functional Properties Differ Between (α4β2)_2_α5^N398^ and (α4β2)_2_α5^D398^ Receptors?

**Figure 2:**
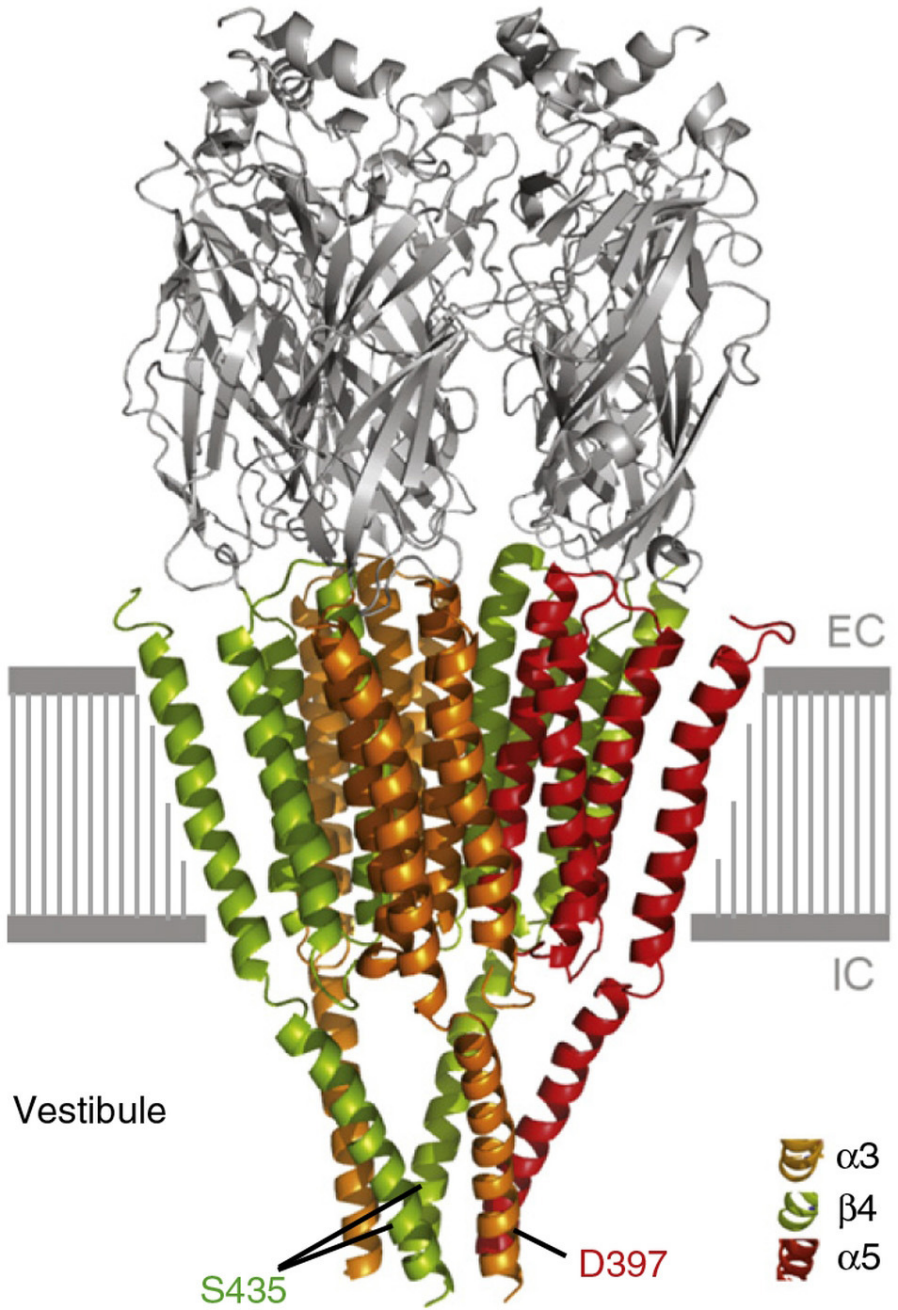
Model of the 3D structure of the (α3β4)_2_α5 nAChR. Transmembrane and intracellular domains of α3, α5, and β4 subunits are shown in orange, red, and green, respectively. The S435 residue in β4 and the D397 residue in α5 are located at the tip of the intracellular vestibule. Note the close apposition between S435 in the β4 subunit and D397 (corresponding to amino acid 398 in the human ortholog) in the α5 subunit. Changing the serine at position 435 in the β4 subunit to the arginine present in the corresponding residue in β2 abolished the β4-specific trafficking of the receptor to the plasma membrane. EC, extracellular space; IC, intracellular space. Reproduced with permission from Frahm et al. (2011).

Kuryatov and colleagues compared the properties of (α4β2)α5 receptors containing either the α5 N398 or D398 variant expressed in *Xenopus* oocytes (Kuryatov et al., 2011). In the α5 subunit, amino acid 398 resides in the large cytoplasmic domain adjacent to the conserved amphipathic α-helix that immediately precedes the fourth transmembrane domain (Figure 2). The authors speculated that in this region, the negatively charged aspartic acid at position 398 in the D398 variant might increase Ca^2+^ permeability, whereas the amide group in the asparagine in the rare N398 variant might reduce Ca^2+^ permeability. Indeed, they found that the N398 α5 variant has significantly lower Ca^2+^ permeability—but similar sensitivity—compared to the D398 variant (Kuryatov et al., 2011). The authors observed no difference in ACh potency between receptors incorporating either the N398 or the D398 α5 variant. However, desensitization in the presence of 3 μM ACh was significantly larger for (α4β2)_2_α5^N398^ than for (α4β2)_2_α5^D398^ receptors, suggesting that the already narrow concentration range for activatable α4β2^*^ receptors relevant at smoking may be further reduced by the N398 variant (“smoldering activation range,” Kuryatov et al., 2011).

In contrast to the tetrameric β2-α4-β2-α4 concatemers with added either N398 or D398 α5, Prevost and colleagues used full pentameric concatemers for expression in *Xenopus* oocytes. Still, no difference was observed in ACh potency, efficacy or acute desensitization when comparing (α4β2)_2_α5^D398^ with (α4β2)_2_α5^N398^ receptors (Prevost et al., 2020). The reduced Ca^2+^ permeability mentioned above explain the smaller Ca^2+^ signal measured using the Ca^2+^-sensing photoprotein aequorin in HEK293T cells transfected with mouse α4, β2, and α5^N397^ subunits^3^, compared to cells transfected with α4, β2, and α5^D397^ subunits (Bierut et al., 2008).

Using qPCR analysis, Oni and colleagues found that *CHRNA3, CHRNA4, CHRNA5, CHRNB2*, and *CHRNB4* mRNA levels (encoding the α3, α4, α5, β2, and β4 nAChR subunits, respectively), were similar in human iPSCs differentiated to primarily dopaminergic neurons, irrespective of whether the *CHRNA5* gene carried the D398 or N398 allele (Oni et al., 2016). Compared to D398-expressing neurons, N398-expressing neurons exhibited greater postsynaptic activity, indicated by the increased frequency and the amplitudes of the spontaneous postsynaptic currents. When the authors differentiated the iPSCs into glutamatergic cells, they found that 0.1 μM nicotine significantly increased the frequency of spontaneous excitatory postsynaptic currents (EPSCs) in neurons carrying the N398 variant, but had no effect in neurons carrying the D398 variant. Higher concentrations of nicotine increased the frequency of EPSCs in neurons carrying the D398 variant, but had no effect in neurons carrying the N398 variant, which could be explained by receptor desensitization at these concentrations. Given the response to sub-micromolar concentrations of nicotine, these differences are likely due to the presence of high-affinity (α4β2)_2_α5 receptors containing either the D398 or N398 α5 variant. Based on gene expression profiling using RNA-seq analysis, the authors speculated that genes specific for the ligand-receptor interaction, Ca^2+^ signaling, and axon guidance are enriched in neurons carrying the N398 α5 variant, thus accounting for the observed differences (Oni et al., 2016). Interestingly, however, the differences observed between neurons carrying the N398 variant and neurons carrying the D398 variant were independent of preceding nAChR activation.

More recently, O'Neill and colleagues found that exposing pregnant mice to nicotine significantly affected the offspring's consumption of nicotine. Nicotine in the drinking water of dames reduced the nicotine consumption of offsprings carrying the α5(D397) gene, whereas consumption was enhanced in offsprings carrying the α5(N397) gene. By examining the underlying cellular and molecular mechanisms, the authors observed that exposure to nicotine during development differentially affected both the function of α4β2^*^ nAChRs in the striatum and the expression of α3β4^*^ nAChRs in the habenula of offsprings (O'Neill et al., 2018).

In VTA slice preparations, application of 100 μM nicotine revealed two distinct neuronal populations; one population responded with a relatively large current of ~40 pA, and the other population responded with a much smaller current (~8 pA). Interestingly, the 40-pA cell population was significantly reduced in mice carrying the N397 α5 variant and missing entirely in α5 KO mice (Sciaccaluga et al., 2015). Furthermore, the number of cultured ventral midbrain cells that responded to 100 μM nicotine, as well as the extent of the increase in intracellular Ca^2+^, was significantly reduced in neurons carrying the N397 variant compared to neurons carrying the D397 variant. Since nicotine application failed to induce an increase in intracellular Ca^2+^ in cultures prepared from α5 KO mice, these observations indicate that the N397 variant can partially substitute for the more common D397 variant in (α4β2)_2_α5 receptors (Sciaccaluga et al., 2015). This notion is supported by experiments with transiently transfected rat pituitary GH4C1 cells, which suggests that the N397 variant can indeed replace the D397 variant; compared to cells expressing (α4β2)_2_β2 receptors, both the current and the number of cells with an increase in intracellular Ca^2+^ in response to 100 μM nicotine were reduced to similar levels in cells expressing either (α4β2)_2_α5^N397^ or (α4β2)_2_α5^D397^ nAChRs (Sciaccaluga et al., 2015).

As discussed above, the firing rates of VIP interneurons and pyramidal cells in layer II/III of the PFC are regulated by α5-containing receptors, likely containing the α4β2^*^ backbone. In α5 KO mice, the reduced firing frequency in these neurons is fully restored by expressing the D397 α5 variant, but is only partially restored by expressing the N397 variant, suggesting that the N397 variant can functionally replace—at least partially—the D397 α5 variant (Koukouli et al., 2017).

In separate experiments, Morel and colleagues used targeted lentiviral infection to express the N397 or D397 α5 variant in α5 KO mice and found that both variants restored DMPP-induced currents in VTA brain slices to the same extent. However, they found that increasing the firing rate of *in vivo* recorded VTA dopaminergic neurons required a 2-fold higher dose of intravenous nicotine in α5 KO mice virally transfected with the N397 α5 variant compared to α5 KO mice transfected with the D397 variant. Together with the previous observation that an 8-fold higher dose of nicotine was required to achieve the same effect in uninfected α5 KO mice, these results indicate that the N397 variant is less potent than the D397 variant at driving nicotine dependence (Morel et al., 2014).

Recently, these findings in mice were confirmed in rats genetically engineered to express the N397 α5 variant on an α5 KO background (Forget et al., 2018). Stimulating nAChRs in VTA brain slices with a saturating concentration of DMPP concentration induced currents that were similar between WT and α5^N397^ rats (Forget et al., 2018). Similarly, and unlike the α5 KO rat, WT and α5^N397^ rats required the same dose of intravenous nicotine in order to increase the firing frequency of VTA neurons. These observations suggest that nAChRs expressed in VTA neurons have similar sensitivity and efficacy regardless of whether they contain the D397 or N397 α5 variant. However, compared to WT rats, the current amplitude measured in IPN neurons stimulated with 30 μM nicotine was significantly reduced in both α5 KO rats and in α5^N397^ rats. Finally, the authors found that α5^N397^ rats self-administered more nicotine at higher doses and exhibited higher levels of nicotine-induced reinstatement of nicotine-seeking behavior compared to WT rats, confirming that the IPN plays a critical role in this behavior (Forget et al., 2018).

In summary (α4β2)_2_α5^N397^ receptors are less sensitive to agonists compared to (α4β2)_2_α5^D397^ receptors. Moreover (α4β2)_2_α5^N398^ receptors have less Ca^2+^ permeability compared to (α4β2)_2_α5^D398^ receptors.

## The α5 Subunit Co-assembles With α3 and β4 in the Central and Peripheral Nervous Systems

### Regional Distribution

Receptors containing α3 and β4 nAChR subunits are predominately expressed in the autonomic nervous system, and the α5 subunits co-assembles with these two subunits to a significant extent (Conroy and Berg, 1995; Mao et al., 2006; David et al., 2010). Moreover, the α3 and β4 subunits are present in several regions in the rodent brain, particularly the MHb and IPN (Sheffield et al., 2000; Grady et al., 2009; Scholze et al., 2012; Beiranvand et al., 2014). In the MHb, α5, although expressed at relatively low levels, co-assembles primarily with α3β4^*^ receptors, as deleting the β4 subunit eliminates all of the [^3^H]-epibatidine-labeled receptors pulled down using an anti-α5 antibody (Scholze et al., 2012). In contrast, mice lacking the β2 subunit have normal levels of α5-containing receptors (Grady et al., 2009; Scholze et al., 2012). Despite this finding, some receptors in the MHb may contain the β2 subunit, as pre-clearing this subunit using an anti-β2 antibody also removes α5-containing receptors in both mice (Scholze et al., 2012) and rats (Grady et al., 2009). Nevertheless, a previous report based on nAChR agonist and antagonist profiling suggested that nAChRs in the rat MHb consist primarily of α3β4^*^ receptors (Quick et al., 1999). Specifically, they found that cytisine was the most efficacious agonist, and the α3β4-specific antagonist α-conotoxin AuIB inhibited ~75% of nicotine-induced currents. Given that the α3β2-specific antagonist α-CtxMII also inhibited currents to a certain extent, the authors proposed that the β2 subunit may contribute to these receptors (Quick et al., 1999), consistent with the above-mentioned immunoprecipitation studies (Grady et al., 2009; Scholze et al., 2012). On the other hand, α5-containing receptors were not detected in the MHb of transgenic α5^GFP^ mice (Hsu et al., 2013), and along with its relatively low expression in the MHb (Morton et al., 2018), *Chrna5* mRNA could not be detected in this brain region using *in situ* hybridization (Wada et al., 1990).

Although the notion that α3β4^*^ receptors are expressed predominantly in the MHb is undisputed, the effect of the α5 accessory subunit on receptor function in the MHb has been a matter of debate (Morton et al., 2018). Transgenic mice overexpressing Chrnb4 exhibit a strong aversion to nicotine, which can be reversed by expressing the N397 α5 variant in the MHb (Frahm et al., 2011). On the other hand, the aversive effects of high nicotine doses on the brain's reward systems are abolished in mice with a targeted loss of α5 subunits, and this effect was reversed by restoring α5 expression in the MHb (Fowler et al., 2011). The converse experiment—knocking down α5 expression using a lentivirus-mediated shRNA injected into the MHb of rats—support the findings in α5 KO mice (Fowler et al., 2011). The authors also found that Fos immunoreactivity (a marker for neuronal activity) was significantly increased in the IPN following an aversively high dose of nicotine in WT mice, but not in α5 KO mice, leading to the conclusion that the α5 subunit has a facilitating effect on receptor function. This effect may be indirect, as nicotine increased the intrinsic excitability of MHb neurons in brain slices, and this increase was mimicked by the application of the neurokinin 1 receptor ligand substance P and the neurokinin 3 receptor agonist neurokinin B, but was prevented by preincubation with the neurokinin 1 receptor antagonist L-732138 and the neurokinin 3 receptor antagonist SB222200, and was absent in α5 KO mice (Dao et al., 2014). Moreover, ^86^Rb efflux induced with 30 μM ACh was significantly reduced in synaptosomes isolated from several brain regions in α5 KO mice, including the habenula and IPN (Fowler et al., 2011). Interestingly, injections of Lenti-*Chrna5* into the MHb attenuated the deficits in ^86^Rb efflux in the IPN, but not in the MHb, of knockout mice.

The IPN receives major cholinergic input from MHb afferents, which release glutamate and ACh (Ren et al., 2011), and studies have shown that the release of ACh from either intact IPN tissue or IPN synaptosomes in response to nAChR activation requires β4-containing receptors (Grady et al., 2009; Beiranvand et al., 2014), suggesting receptors consisting of the α3β4^*^ backbone. The finding that ACh release was also reduced in β3 KO mice suggests that β3β4^*^ receptors also contribute to this release (Grady et al., 2009). On the other hand, the agonist-induced release of ACh is not facilitated by α4, α5, or β2 subunits (Grady et al., 2009; Beiranvand et al., 2014). With respect to norepinephrine release from the IPN and MHb, however, the concentration-response curves depended on β2-containing receptors; moreover, the curves were right-shifted in α5 KO mice, and norepinephrine release was abolished in the presence of TTX (Beiranvand et al., 2014).

In the autonomic nervous system, α3β4^*^ receptors form the predominant receptor backbone, unlike in the CNS. In both mouse and rat ganglia, ~25% of these receptors contain the α5 subunit (Mandelzys et al., 1994; Mao et al., 2006; Putz et al., 2008; David et al., 2010). Given the finite number of possibly nAChR subunit combinations, and given the availability of various mouse—and more recently, rat—KO models, the ganglia in the autonomic nervous system are an ideal system for studying the composition and function of specific endogenous nAChRs.

In summary, the α5 subunit co-assembles with the α3 and β4 subunits throughout the autonomic nervous system. However, nAChRs in the medial habenula contain (α3β4)_2_α5 receptors at low levels.

### Receptor Affinity and Efficacy

Like α4β2^*^ receptors discussed above, the properties of α3β4^*^ receptors, and how these properties are affected by the presence of the α5 subunit, have been studied in heterologous expression systems. For example, Wang and colleagues reported no significant difference in the potency or efficacy of ACh and nicotine between human α3β4^*^ receptors lacking the α5 subunit and (α3β4)_2_α5 receptors when expressed in *Xenopus* oocytes (Wang et al., 1996). On the other hand, expressing α5 with α3 and β4 receptors at a 1:1:1 ratio in *Xenopus* oocytes significantly increased Ca^2+^ permeability and the rate of receptor desensitization (Gerzanich et al., 1998). These observations were later confirmed by Groot-Kormelink and colleagues, who also observed an increase in apparent receptor desensitization and no difference in ACh potency or efficacy between α3β4^*^ and (α3β4)_2_α5 receptors expressed in *Xenopus* oocytes; to maximize the number of (α3β4)_2_α5 receptors, the α5, α3, and β4 subunits were expressed at a ratio of 20:1:1 (Groot-Kormelink et al., 2001). In a separate study in which *Xenopus* oocytes expressed mouse α3 and β4 subunits (at a 1:1 ratio), ACh produced a monophasic concentration-response curve when peak current was measured, but a biphasic curve when net charge was measured, suggesting the expression of both high-affinity and low-affinity receptors; the addition of α5 expression (at a 1:1:1 ratio with α3 and β4) had no effect on the ACh EC_50_ value for peak current, but produced a monophasic ACh concentration-response curve for net charge, consistent with the hypothesis that α5 co-expression shifts receptors toward a single α5-containing form (Papke et al., 2010). In follow-up experiments by the same group, none of the agonists studied, including ACh, nicotine, cytisine, and varenicline, differed in potency between human (α3β4)_2_β4 and (α3β4)_2_α5 receptors expressed in *Xenopus* oocytes by injecting the dimeric α3-β4 concatemer together with either β4 or α5 (Stokes and Papke, 2012).

Similarly, no significant difference was found with respect to the potency of ACh, nicotine, or cytisine among (α3β4)_2_β4, (α3β4)_2_α3, and (α3β4)_2_α5 receptors expressed as pentameric concatemers in *Xenopus* oocytes, although the nAChR agonists ACh, nicotine, and cytisine had higher efficacy in α5-containing receptors compared to α3- and β4-containing receptors (George et al., 2012). In contrast, when α3 and β4 were injected separately, the addition of α5 at a ratio of 1:1:1 significantly reduced the expression of functional receptors, possibly due to an adverse effect of separate α5 subunits with respect to forming functional nAChRs (George et al., 2012). Interestingly, the potency of mecamylamine decreased by an order of magnitude between (α3β4)_2_β4 and (α3β4)_2_α5 receptors, regardless of whether they were expressed as separate subunits or as concatemers (George et al., 2012). These observations are similar to results obtained regarding the effect of hexamethonium on inhibiting transganglionic transmission in the superior cervical ganglion (SCG) of WT mice and α5β2 KO mice (i.e., expressing α3β4^*^ receptors), with IC_50_ values of 389.2 and 126.7 μM, respectively (Simeone et al., 2019). By taking into consideration the contribution of (α3β4)_2_β4 receptors in WT mice, the authors calculated an IC_50_ of 568.6 μM for (α3β4)_2_α5 receptors (Simeone et al., 2019).

Studies have found no difference in the potency or efficacy of agonists between α3β4^*^ receptors expressed either with or without the α5 subunit in tsA201 cells (Wang et al., 1998; Nelson et al., 2001). The presence of the α5 subunit also had no effect on the decay of currents elicited by 300 μM ACh (Wang et al., 1998). However, immunoprecipitation studies using a cell line expressing (α3β4)_2_α5 receptors showed that only 14% of all nAChRs contained the α5 subunit (Wang et al., 1998; Nelson et al., 2001), which may be too low to reveal any meaningful effect of the α5 subunit. To overcome this issue, Li and colleagues transiently transfected α3β4-expressing HEK293 cells with a FLAG-tagged α5 subunit, allowing them to selectively study cells that bound to small beads coated with an anti-FLAG antibody (Li et al., 2011). Using this strategy combined with patch-clamp recording, the authors found that the presence of the α5 subunit had no effect on the potency of ACh, nicotine, cytisine, or DMPP (Li et al., 2011). In BOSC-23 cells (a human kidney cell line derived from the 293T cell line), co-expressing the α5 subunit with α3β4 receptors caused a significant right-shift in the ACh concentration-response curve, as well as a reduction in the peak amplitude of the response; in contrast, when α5 was co-expressed with α3 and β4 in *Xenopus* oocytes, no observable difference was found with respect to ACh potency or efficacy (Fucile et al., 1997).

Decreased agonist efficacy in the presence of the α5 subunit was also observed when receptor function was measured using the bioluminescent Ca^2+^ indicator aequorin. At saturating concentrations of the agonists nicotine and varenicline, together with 20 mM Ca^2+^ in the recording solution, the increase in intracellular Ca^2+^ concentration was significantly higher in HEK293 cells stably expressing α3β4^*^ compared to cells expressing (α3β4)_2_α5; importantly, this effect of the α5 subunit was not due to a change in either the total or surface expression of the receptors (Tammimaki et al., 2012). Interestingly, and in contrast to results obtained with *Xenopus* oocytes (George et al., 2012), Tammimaki and colleagues found that the potency of mecamylamine was somewhat higher in cells expressing the α5 subunit (Tammimaki et al., 2012). Recently, the reduced efficacy of nicotine at activating (α3β4)_2_α5 receptors compared to (α3β4)_2_β4 receptors was confirmed by measuring aequorin luminescence in HEK293 cells stably transfected with human α3 and β4 subunits and cells stably expressing α3, β4, and α5 subunits (Ray et al., 2017).

Yu and Role studied the effect of α5 on α3β4^*^ receptors in chick sympathetic neurons by functionally deleting the α5 subunit with antisense oligonucleotide treatment. The deletion of α5 significantly increased the potency of both ACh and cytisine. ACh was more efficacious than cytisine both with and without antisense treatment, but the difference was significantly larger upon deletion of α5. As deletion of α5 also eliminated channels that were blocked by the α7-specific antagonist methyllycaconitine while increasing the percentage of current carried by nAChRs that are sensitive to α-bungarotoxin, the authors inferred that native sympathetic neurons express heteromeric nAChRs that include both α5 and α7 (Yu and Role, 1998).

In β2 KO mice, the SCG neurons express ~75% α3β4^*^ receptors and ~25% (α3β4)_2_α5 receptors, whereas α5β2 double-KO mice express exclusively α3β4^*^ hetero-pentameric receptors, making these ideal models for investigating the role of the α5 subunit in endogenous α3β4^*^ receptors (David et al., 2010). Moreover, as discussed below, experiments showed that the percentage of (α3β4)_2_α5 receptors in the plasma membrane is significantly higher than the percentage of the overall receptor pool determined by immunoprecipitation (Simeone et al., 2019). With respect to ligand potency, David and colleagues found no difference in either the potency or efficacy of cytisine or DMPP between cultured neurons prepared from either α5β2 double-KO mice or β2 KO mice, and the total number of nACh receptors was not reduced in either α5β2 double-KO mice or β2 KO mice (David et al., 2010). With respect to the single-channel properties, unitary conductance was similar between α5-containing receptors and α3β4^*^ hetero-pentameric receptors; however, α5-containing receptors had a longer open dwell time and longer burst duration (Ciuraszkiewicz et al., 2013).

In cultured SCG neurons, both α3β4^*^ hetero-pentameric receptors and (α3β4)_2_α5 receptors are present at presynaptic sites, where receptor activation by ACh, nicotine, cytisine, DMPP, or epibatidine in the presence of TTX (for blocking voltage-gated Na^+^ channels) or Cd^2+^ (for blocking voltage-gated Ca^2+^ channels) triggers the release of preloaded [^3^H]-norepinephrine (Kristufek et al., 1999; Fischer et al., 2005b). Although agonist potency differed slightly between SCG cultures prepared from α5 KO mice and WT mice, the agonists' efficacy was considerably higher in α5 KO neurons (Fischer et al., 2005b). Importantly, the release of [^3^H]-norepinephrine required extracellular Ca^2+^ in the superfusion buffer, suggesting that Ca^2+^ entry via nAChRs triggers exocytosis and transmitter release, and intracellular Ca^2+^ imaging revealed that nAChR agonists induce a larger Ca^2+^ transient in α5 KO neurons compared to WT neurons (Fischer et al., 2005b). Given that the peak current amplitudes elicited in response to saturating concentrations of ACh, DMPP, and cytisine were similar between α3β4^*^ hetero-pentameric receptors and (α3β4)_2_α5 receptors (David et al., 2010), the above-mentioned findings suggest that receptors lacking the α5 subunit have increased Ca^2+^ permeability, increased Ca^2+^ release from intracellular stores, or an increase in additional downstream mechanisms, rather than a general increase in receptor efficacy. These findings are reminiscent of the results obtained using HEK293 cells expressing α3 and β4 subunits vs. cells expressing α3, β4, and α5 subunits (Tammimaki et al., 2012; Ray et al., 2017).

In summary, the α5 subunit does not significantly affect the sensitivity of α3β4^*^ receptors expressed with concatemer constructs in *Xenopus* oocytes. However, heterologously expressed human receptors containing the α5 subunit are activated at higher efficacy than either (α3β4)2α3 or (α3β4)2β4 receptors. In contrast, endogenous receptors recorded in SCG neurons in α5 KO mice do not differ significantly from WT neurons with respect to either activation sensitivity or efficacy. Activation of α3β4^*^ leads to a significantly higher increase in intracellular Ca^2+^ compared to (α3β4)_2_α5 receptors expressed in HEK293 cells. Likewise, SCG neurons taken from α5 KO mice show a significantly higher increase in intracellular Ca^2+^ upon receptor activation compared SCG neurons taken from WT mice, and receptor activation also induces a significantly higher release of preloaded [^3^H]-norepinephrine in SCG neurons taken from α5 KO mice. This contrasts observations in *Xenopus* oocytes, where (α3β4)_2_α5 receptors have higher Ca^2+^ permeability compared to α3β4^*^ receptors.

### Desensitization Properties

When expressed in *Xenopus* oocytes, the addition of the α5 subunit to the α3 and β4 subunits increased the rate of apparent receptor desensitization during ACh application (Gerzanich et al., 1998; Groot-Kormelink et al., 2001). Indeed, expressing the α5 subunit in a tsA201 cell line stably expressing human α3β4 receptors had no effect on the decay of currents elicited in response to 300 μM ACh (Nelson et al., 2001). Similarly, no difference in decay was observed using a higher concentration of ACh (1 mM), whereas 100 μM nicotine prolonged the current decay in HEK293 cells transfected with α5, α3, and β4 subunits compared to cells expressing only the α3 and β4 subunits (Li et al., 2011).

When HEK293 cells expressing either α3β4^*^ or α3β4α5 receptors were incubated for 30 s with 1 mM ACh or 100 μM nicotine, the time course of recovery from desensitization measured using patch-clamp recording was similar between the two receptor subtypes (Li et al., 2011). Moreover, using the intracellular Ca^2+^-sensing photoprotein aequorin, Tammimaki and colleagues found no significant difference in nicotine IC_50_ values between α3β4^*^ and (α3β4)_2_α5 receptors stably expressed in HEK293 cells upon prolonged exposed to nicotine (Tammimaki et al., 2012).

The current decay measured in response to 300 μM ACh and fit with the sum of two exponential functions was similar between α5 single-KO and α5β2 double-KO mice, which express α3β4^*^ hetero-pentameric receptors and (α3β4)_2_α5 receptors, respectively (David et al., 2010). These KO mouse models were also used to measure receptor desensitization during prolonged exposure to nicotine in an intact SCG preparation in which compound action potentials (CAPs) were recorded from the postganglionic nerve in response to supramaximal stimulation of the preganglionic nerve. Nicotine added to the superfusion buffer at increasing concentrations yielded IC_50_ values of 3.01 μM for α3β4^*^ receptors and 3.67 μM for (α3β4)_2_α5 receptors (Simeone et al., 2019). This small, yet statistically significant difference indicates that the presence of the α5 subunit provides a slight degree of protection from receptor desensitization, although these levels of nicotine are not achieved, even with heavy smoking (Moreyra et al., 1992).

Varying the stimulation frequency of the preganglionic nerve also revealed differences in CAP amplitude between ganglia expressing α3β4^*^ receptors and ganglia expressing (α3β4)_2_α5 receptors (Simeone et al., 2019). With a stimulation frequency of 5 Hz, CAP amplitude increased significantly in WT ganglia but not in α5β2 KO ganglia; with 10-Hz stimulation, however, CAP amplitude decreased in α5β2 KO ganglia but not in WT ganglia, suggesting differences in activation and/or desensitization properties between these two receptors (Simeone et al., 2019).

In summary, the α5 subunit has just minor effects, if at all, on the desensitization properties of α3β4^*^ receptors.

### Receptor Expression and Membrane Trafficking

Measuring the total number of receptors using methods such as radiolabeled ligands (Brown et al., 2007; Baddick and Marks, 2011) or immunoprecipitation of solubilized [^125^I]-epibatidine–labeled receptors (Tammimaki et al., 2012) does not necessarily provide information regarding the number of functional receptors that actually reach the cell surface, requiring morphological, biochemical, and/or functional techniques in order to monitor receptor trafficking to the plasma membrane. Studies have shown that a number of chaperone proteins and regulatory proteins play a role in the trafficking of homo- and hetero-pentameric nAChR receptors to the plasma membrane (Millar and Harkness, 2008; St John, 2009; Crespi et al., 2018a). Because heterologous expression systems provide a robust model for studying receptor processing and trafficking, our current knowledge regarding the role of the α5 subunit in these processes stems primarily from studies involving cell lines (e.g., HEK293 cells), normal rat kidney cells, and *Xenopus* oocytes.

Combining [^125^I]-mAb210 immunolabeling, two-electrode voltage clamp, and single-channel electrophysiology, George and colleagues reported differential effects of lynx1 on surface expression and functional properties of (α3β4)_2_α3, (α3β4)_2_β4, and (α3β4)_2_α5 in oocytes (George et al., 2017). Lynx family proteins are related to elapid snake venom toxin genes, such as α-bungarotoxin, consisting of a three-fingered folding motif and multiple internal disulfide bonds (Miwa et al., 2019). Lynx1 was previously shown to directly bind to α4β2^*^ nAChRs (Ibanez-Tallon et al., 2002) and to shift nAChR stoichiometry to low sensitivity (α4β2)_2_α4 pentamers (Nichols et al., 2014). Lynx1 reduced (α3β4)_2_β4 nAChR function principally by lowering cell-surface expression, whereas decreased unitary conductance, enhanced closed dwell times, and reduction in the proportion of long bursts accounted for reduced function of (α3β4)_2_α3 receptors. Alterations in both cell-surface expression and single-channel properties mediated by lynx1 accounted for the reduction in (α3β4)_2_α5 function (George et al., 2017).

Using [^125^I]-epibatidine binding of solubilized receptors and an mAb35-based ELISA assay in intact cells, Tammimaki and colleagues found no difference in the number of receptors between HEK293 cells stably expressing α3 and β4 subunits and cells expressing α3, β4, and α5 subunits (Tammimaki et al., 2012), suggesting that the α5 subunit does not interfere with the assembly and membrane trafficking of these receptors. In contrast, Ray and colleagues reported that co-expressing the human α5 subunit significantly reduced the number of α3β4^*^ receptors at the plasma membrane in HEK293 cells (Ray et al., 2017). For their experiments, the authors inserted an N-terminal HA, cMYC, and V5 tag in the α3, β4, and α5 subunits, respectively, finding that 98% of receptors were (α3β4)_2_β4 receptors in cells expressing α3 and β4, whereas 50% of the receptors were (α3β4)_2_β4 and 50% were (α3β4)_2_α5 receptors in cells expressing α3, β4, and α5 subunits (Ray et al., 2017). Strikingly different results were obtained in rat kidney cells co-transfected with a dimeric plasmid expressing α3β4 together with a plasmid expressing α3, β4, or α5 (Crespi et al., 2018b). Using this approach, the authors found that including the β4-expressing construct resulted in (α3β4)_2_β4 receptors that reached the plasma membrane; in contrast, combining the dimeric construct with α3 resulted in (α3β4)_2_α3 receptors that failed to exit the endoplasmic reticulum, and combining the dimeric construct with α5 resulted in (α3β4)_2_α5 receptors were unable to exit the Golgi apparatus and were shuttled back to the endoplasmic reticulum (Crespi et al., 2018b).

An indirect method for measuring nAChRs at the cell surface is to record whole-cell currents in response to a saturating concentration of agonists; this approach has been used to determine whether the α5 subunit plays a role in receptor trafficking to the plasma membrane (e.g., Frahm et al., 2011; George et al., 2012). Using 2-electrode voltage-clamp recordings in *Xenopus* oocytes, Frahm and colleagues identified structural components in nAChR subunits critical for the trafficking of α3β4^*^ receptors to the plasma membrane (Frahm et al., 2011). They found that injecting mouse β4—but not β2—cRNA in excess of α3 cRNA significantly increased currents elicited by 100 μM nicotine; if they included an excess amount α5 cRNA, nicotine-induced currents were reduced. They also found that the apparent potentiating effect of β4 was specific to amino acid S435, as it was eliminated by changing this serine to an arginine, the amino acid present in the equivalent position (R431) in the β2 subunit. Conversely, expressing a β2 subunit with a serine at position 431 results in a potentiation of nicotine-induced currents, giving the β2 subunit “β4-like” properties (Frahm et al., 2011). These results may explain the finding that the total number of nAChRs in the SCG is reduced by ~90% in β4 KO mice, while deleting the β2 subunit has no effect (David et al., 2010). Homology modeling by Frahm and colleagues using the *Torpedo* nAChR suggests that the receptor's five subunits form a vestibule in which residue S435 in the β4 subunit is in apposition to residue D397—corresponding to residue 398 in the human ortholog—in the α5 subunit (Figure 2) (Frahm et al., 2011). Notably, when expressed in *Xenopus* oocytes, the α5 subunit reduced receptor trafficking only when the non-concatenated forms of human α3 and β4 cRNA were used; expressing the α5 subunit had no effect when concatenated α3-β4 cRNA was used (George et al., 2012).

Interestingly, SCG neurons obtained from α5 KO mice did not differ from WT neurons with respect to the total number of receptors measured using immunoprecipitation or peak currents measured in response to ACh, cytisine, or DMPP, suggesting that the α5 subunit does not affect expression and membrane trafficking of endogenous α3β4^*^ receptors (David et al., 2010). Conversely, the authors found that β4 KO mice do not express α5-containing receptors, with the small number of remaining receptors (corresponding to ~10% of the number of receptors measured in WT mice) comprised of α3β2^*^ receptors (David et al., 2010). This finding differs from several heterologous systems, in which the receptors can be “forced” into an (α3β2)_2_α5 configuration (Wang et al., 1996, 1998; Fucile et al., 1997; Nelson et al., 2001; Papke et al., 2010).

As noted above, only 25% of all receptors in the SCG of β2 KO mice contain the α5 subunit, and this relatively small contribution to the total receptor pool may not be sufficient to reveal differences at the whole-cell level. However, the non-competitive nAChR antagonist hexamethonium was significantly less potent at blocking (α3β4)_2_α5 receptors compared to the α3β4^*^ hetero-pentameric receptors expressed in α5β2 double-KO mice (see also Wang et al., 2002; Simeone et al., 2019). Using the difference in the right-shift in the concentration-response curve allowed Simeone and colleagues to calculate the potency of hexamethonium at blocking a hypothetical “pure” population of (α3β4)_2_α5 receptors, as well as the percentage of these receptors present at the cell surface. Interestingly, the authors found that hexamethonium inhibited transganglionic transmission in intact ganglia to the same extent as cultured SCG neurons stimulated with 100 μM ACh, suggesting that α5-containing receptors are dispersed along the surface of SCG neurons and are not specifically targeted to synaptic sites (Simeone et al., 2019). The finding that 72 and 63% of surface receptors are (α3β4)_2_α5 receptors in intact ganglia and cultured SCG neurons, respectively, indicates that α5-containing receptors are enriched at the plasma membrane (Simeone et al., 2019).

In summary, the majority of studies show that the α5 subunit does not significantly affect either the total number of receptors or the number of receptors expressed at the plasma membrane.

### Do the Functional Properties Differ Between (α3β4)_2_α5^N398^ and (α3β4)_2_α5^D398^ Receptors?

In *Xenopus* oocytes, the agonists ACh, nicotine, cytisine, and varenicline had similar potencies at activating (α3β4)_2_α5^D398^ receptors vs. (α3β4)_2_α5^N398^ receptors expressed by injecting the α3-β4 concatemer together with the respective α5 variant (Stokes and Papke, 2012). Similarly, Kuryatov and colleagues found no difference with respect to ACh EC_50_ values, Ca^2+^ permeability, or short-term desensitization in response to a 3-s exposure to 100 μM ACh when separately injecting α3, β4, and either α5^D398^ or α5^N398^ cRNA at a ratio of 1:1:2 in *Xenopus* oocytes (Kuryatov et al., 2011).

As discussed above, nAChR expression in *Xenopus* oocytes can be significantly increased by injecting an excess amount of mouse β4 cRNA compared to α3 cRNA. However, this increased expression can be reduced by co-injecting α5 cRNA, suggesting that this residue in the α5 subunit may play a role in receptor assembly, processing, and/or trafficking. Moreover, when α3, β4, and α5 cRNA was injected at a 1:10:10 ratio, the N397 α5 variant was significantly more effective at decreasing receptor expression compared to the D397 variant (Frahm et al., 2011). Consistent with this finding, George and colleagues found that receptors expressed using the fully pentameric β4-α3-β4-α3-α5 concatemer differed significantly in their ACh-induced peak currents depending on whether the α5 subunit was the N398 or D398 variant (George et al., 2012). Still, none of the nAChR ligands tested differed in potency between the two α5 variants, regardless of whether the receptors were expressed using concatemers or separate constructs (George et al., 2012).

By taking advantage of the fusion proteins encoding concatemers of human α3, β4, α5^D398^, and α5^N398^ subunits, Ochoa and colleagues tested the effects of the co-expressed protoxin LYPD6B on distinct nAChRs in *Xenopus* oocytes. LYPD6B enhanced ACh potency for (α3β4)_2_α3 while reducing efficacy for (α3β4)_2_α3 and (α3β4)_2_α5^D398^ receptors, whereas the properties (α3β4)_2_β4 and (α3β4)_2_α5^N398^ remained unaffected (Ochoa et al., 2016).

Interestingly, both the peak current amplitude and the EC_50_ values for nicotine and acetylcholine were slightly but significantly higher in human iPSC-derived dopaminergic neurons carrying the N398 α5 variant compared to cells carrying the D398 variant (Deflorio et al., 2016). However, RT-PCR analysis revealed that these cells express both α4β2^*^ and α3β4^*^ receptors (Deflorio et al., 2016), suggesting that the polymorphism may have affected α4β2^*^ as well as α3β4^*^receptors, although the ACh EC_50_ values of 63.4 μM (in cells with the D398 variant) and 93.9 μM (in cells with the N398 variant) suggest that the difference in currents primarily reflected the properties measured for the low-affinity α3β4^*^ receptors.

Using patch-clamp recording, Li and colleagues found no significant differences in the functional properties (e.g., agonist potency and efficacy, receptor desensitization, or time course of recovery from desensitization) between α3 and β4 subunits co-expressed in HEK293 cells with either the D398 or N398 α5 variant (Li et al., 2011). In contrast, Tammimaki and colleagues measured changes in intracellular Ca^2+^ using aequorin and found significant differences between HEK293 cells expressing (α3β4)_2_α5^N398^, (α3β4)_2_α5^D398^, and α3β4^*^ receptors, with cells expressing α3β4^*^ receptors having the highest response (Tammimaki et al., 2012). In these cells, IP_3_ receptors and ryanodine receptors contribute to the increase in intracellular Ca^2+^, although the role of IP_3_ receptors was larger in the two cell lines expressing (α3β4)_2_α5 receptors compared to the cell line expressing α3β4^*^ receptors; paradoxically, however, the cell lines expressing α3β4^*^ receptors had the largest agonist-induced increase in intracellular Ca^2+^ (Tammimaki et al., 2012).

Recently, Ray and colleagues performed a comprehensive directed screen of a large library of potential nAChR ligands and small molecule kinase inhibitors in order to identify candidate compounds that can distinguish between (α3β4)_2_β4, (α3β4)_2_α5^D398^, and (α3β4)_2_α5^N398^ receptors. The authors used aequorin to measure the effect of 100 μM nicotine in HEK293 cell lines stably expressing the various receptors, finding 8 antagonists that differed between the various receptors, with some compounds able to distinguish between (α3β4)_2_α5^D398^ and (α3β4)_2_α5^N398^ receptors (Ray et al., 2017). Moreover, some molecules such as the EGF receptor tyrosine kinase inhibitor tyrphostin-25 and Zaprinast, an inhibitor of cGMP-specific phosphodiesterases V an VI, prevented the nicotine-induced increase in intracellular Ca^2+^ in cells expressing (α3β4)_2_β4 receptors, but had no effect in cells expressing either (α3β4)_2_α5^D398^ or (α3β4)_2_α5^N398^ receptors (Ray et al., 2017). Finally, the authors found that some molecules such as the protein tyrosine kinase inhibitor genistein and the protein kinase C inhibitor hypocrellin A differentially affected cells expressing (α3β4)_2_α5^D398^ vs. (α3β4)_2_α5^N398^ receptors (Ray et al., 2017).

In summary (α3β4)_2_α5^D398^ and (α3β4)_2_α5^N398^ receptors have similar properties with respect to sensitivity and efficacy; in contrast, these receptors differ significantly with respect to the effect of specific kinase inhibitors.

## Summary and Perspectives

### (α4β2)_2_α5 Receptors

A growing body of evidence based on both heterologously expressed and endogenous nAChR subunits indicates that the addition of the α5 subunit to α4β2^*^ receptors significantly increases the receptor's Ca^2+^ permeability; for example, assays that measure intracellular Ca^2+^ have shown an increased efficacy of (α4β2)_2_α5 receptors compared to α4β2^*^ receptors. This increased permeability affects downstream Ca^2+^-dependent signaling, including nAChR-mediated transmitter release. Studies using electrophysiology have also shown that native (α4β2)_2_α5 receptors have increased efficacy compared to α4β2^*^ receptors.

The ligand affinity of (α4β2)_2_α5 receptors is similar to high-affinity (α4β2)_2_β2 receptors. Thus, replacing low-affinity (α4β2)_2_α4 receptors with (α4β2)_2_α5 receptors in a mixed population containing both (α4β2)_2_α4 and (α4β2)_2_β2 receptors will result in an overall population consisting of highly sensitive (i.e., high-affinity) receptors. In addition, the presence of the α5 subunit “protects” α4β2^*^ receptors from chronic desensitization in the prolonged presence of even low concentrations of nicotine. In various heterologous expression systems, although the number of α4β2^*^ receptors may be increased by expressing the α5 subunit, membrane trafficking of the resulting (α4β2)_2_α5 receptors may be reduced, leading to fewer receptors at the cell surface.

Most—but not all—cellular assays suggest that (α4β2)_2_α5 receptors containing the N398 α5 variant may have reduced functionality (i.e., reduced sensitivity and/or efficacy) compared to receptors containing the D398 variant, but increased functionality compared to α4β2^*^ hetero-pentameric receptors. Thus, the N398 α5 variant appears to be able to replace—at least partially—the D398 variant in α4β2^*^ receptors. Moreover, neither knocking out the α5 subunit nor replacing the D398 variant with the N398 variant significantly affects the overall expression of nAChRs in the CNS. Although recent studies involving both mice and rats have shown differences in drug-seeking behavior between WT animals (i.e., carrying the D398 variant) and animals carrying the N398 variant, the underlying cellular mechanisms remain poorly understood and warrant future study.

### (α3β4)_2_α5 Receptors

In various heterologous expression systems, the presence of the α5 subunit has been found to increase, reduce, or have no effect on the number of α3β4^*^ receptors, depending on the expression system used. In sympathetic neurons in α5 KO mice, loss of the α5 subunit does not affect currents induced by saturating concentrations of agonists, suggesting that the α5 subunit does not affect the number of functional receptors that traffic to the plasma membrane in these neurons. However, studies have shown that the α5 subunit requires the β4 subunit for proper expression of endogenous receptors, as β4 KO mice lack α5-containing receptors. Although (α3β4)_2_α5 receptors do not differ significantly from α3β4^*^ hetero-pentameric receptors with respect to agonist potency or desensitization, nAChR antagonists such as mecamylamine and hexamethonium can distinguish between α3β4^*^ and (α3β4)_2_α5 receptors.

Interestingly, the increase in intracellular Ca^2+^ upon receptor activation is differentially affected by the addition of the α5 subunit to α4β2^*^ vs. α3β4^*^ receptors. With respect to α3β4^*^ receptors, addition of the α5 subunit decreases the Ca^2+^ response, despite the paradoxical finding that α5-containing receptors have increased Ca^2+^ permeability measured using voltage-clamp recordings in *Xenopus* oocytes. Thus, compared to (α3β4)_2_α5 receptors, activating α3β4^*^ hetero-pentameric receptors cause a larger increase in intracellular Ca^2+^ and increased transmitter release. A review of the published literature suggests that this increase is unlikely to be due solely to an increased number of receptors at the plasma membrane when α5 is knocked out. Finally, evidence suggests that (α3β4)_2_α5 receptors containing the N398 α5 variant are more effective than receptors containing the D398 variant with respect to preventing the increase in intracellular Ca^2+^.

### Perspectives

The growing list of compounds that can distinguish between α3β4^*^, (α3β4)_2_α5^D398^, and/or (α3β4)_2_α5^N398^ receptors based on inhibiting the receptor directly or inhibiting downstream signaling provide a robust set of tools for studying how the α5 subunit—and its two variants—affects the function of α4β2^*^ and α3β4^*^ receptors. A key to resolving the underlying mechanisms may be the intracellular loop connecting the third and fourth transmembrane domains, the site in which where nAChR subunits have the highest diversity, containing putative phosphorylation and protein-binding sites. As eloquently summarized by Stokes and colleagues (p. 522), “if we want insights into the functional roles of specific nAChR subtypes, we will have to make efforts to reveal the hidden functions of their intracellular domains” (Stokes et al., 2015).

## Author Contributions

Both authors contributed to the writing of this review.

## Conflict of Interest

The authors declare that the research was conducted in the absence of any commercial or financial relationships that could be construed as a potential conflict of interest.

## Footnotes

^1^The asterisk denotes that the two subunits listed (e.g., α4 and β2) comprise the backbone, with an additional subunit completing the pentamer. The additional subunit can be an “accessory” subunit such as α5 or a “complementary” subunit such as α4 or β2, giving rise to (α4β2)_2_α4 or (α4β2)_2_β2 pentamers, respectively.

^2^Thalamic neurons in WT mice express (α4β2)_2_α4, (α4β2)_2_β2, and (α4β2)_2_α5 receptors.

^3^In the mouse and rat homologs, amino acid 397 corresponds to amino acid 398 in the human α5 protein.

## Abbreviations

ACh: acetylcholine
α-CtxMII: α-conotoxin MII
CAP: compound action potential
CNS: central nervous system
DhβE: dihydro-β-erythroidine
DMPP: dimethylphenylpiperazinium
EPSC: excitatory postsynaptic current
EPSP: excitatory postsynaptic potential
HEK: human embryonic kidney
IPN: interpeduncular nucleus
iPSC: induced pluripotent stem cell
KO: knockout
MHb: medial habenula
nAChR: nicotinic acetylcholine receptor
PFC: prefrontal cortex
PNS: peripheral nervous system
SCG: superior cervical ganglion
SNP: single-nucleotide polymorphism
TTX: tetrodotoxin
VIP: vasoactive intestinal polypeptide
VTA: ventral tegmental area
WT: wild-type.
